# A miRNA-101-3p/Bim axis as a determinant of serum deprivation-induced endothelial cell apoptosis

**DOI:** 10.1038/cddis.2017.219

**Published:** 2017-05-18

**Authors:** Ji-Hee Kim, Dong-Keon Lee, Joohwan Kim, Seunghwan Choi, Wonjin Park, Kwon-Soo Ha, Tae-Hoon Kim, Jongseon Choe, Moo-Ho Won, Young-Guen Kwon, Young-Myeong Kim

**Affiliations:** 1 Department of Molecular and Cellular Biochemistry, School of Medicine, Kangwon National University, Chuncheon, Gangwon-do 24341, Republic of Korea; 2 Department of Immunology, School of Medicine, Kangwon National University, Chuncheon, Gangwon-do 24341, Republic of Korea; 3 Department of Neurobiology, School of Medicine, Kangwon National University, Chuncheon, Gangwon-do 24341, Republic of Korea; 4 Department of Biochemistry, College of Life Science and Biotechnology, Yonsei University, Seoul 03722, Republic of Korea

## Abstract

Serum deprivation or withdrawal induces apoptosis in endothelial cells, resulting in endothelial cell dysfunction that is associated with cardiovascular disease. However, there is still limited information on the role of miRNA in serum deprivation-induced apoptosis. Here we found that serum deprivation increased caspase-dependent apoptosis through miRNA-101-3p downregulation, without altering expression of its host gene RNA 3′-terminal phosphate cyclase-like 1, which was highly correlated with suppressed expression levels of Dicer and Argonaute 2 (Ago2), indicating that miR-101-3p is post-transcriptionally elevated in serum-deprived conditions. The decreased miR-101-3p caused elevated Bim expression by targeting its 3′-untranslated region (3′-UTR). This resulted in activation of the intrinsic pathway of apoptosis via interaction with Bcl-2, decreased mitochondrial membrane potential, cytochrome *c* release, mitochondrial reactive oxygen species (ROS) production, and caspase activation. These events were abrogated by miR-101-3p mimic and the proapoptotic Bim siRNA, which suggest a determinant role of the miR-101-3p/Bim axis in serum deprivation-induced apoptosis. The apoptosis induced by miR-101-3p-mediated Bim expression is mediated by both caspase-3 and -1, which are activated by two distinct intrinsic mechanisms, cytochrome *c* release and ROS-induced inflammasome activation, respectively. In other words, the antioxidant inhibited endothelial cell death mediated by caspase-1 that activated caspase-7, but not caspase-3. These findings provide mechanistic insight into a novel function of miR-101-3p in serum withdrawal-induced apoptosis triggered by activating two different intrinsic or mitochondrial apoptosis pathways, implicating miR-101-3p as a therapeutic target that limits endothelial cell death associated with vascular disorders.

Dysfunction of endothelial cells is directly associated with impaired vasorelaxation, increased inflammation, and increased migration and proliferation of smooth muscle cells.^1^ As a result, endothelial cell apoptosis, an important marker of vascular damage, is critically implicated in the pathogenesis of various cardiovascular diseases including atherosclerosis, hypertension, heart attack, and stroke.^2^

On the basis of mechanisms leading to distinct morphologies, cell death types are classified into apoptosis, autophagic cell death, necroptosis, pyroptosis, and necrosis.^3, 4^ Among them, apoptosis is caused by two distinct signaling cascades, the extrinsic and intrinsic pathways. The extrinsic pathway triggered by death receptor activation is well characterized by identified intracellular signal cascades and sequential caspase activation.^5^ In contrast, the intrinsic pathway in response to cellular stresses, such as hypoxia, growth factor deprivation, and cytotoxic chemicals, activates caspase-9 via the release of mitochondrial cytochrome *c*.^6^ The mitochondrial pathway is primarily regulated by the relative levels and activity of the antiapoptotic and proapoptotic Bcl-2 family proteins.^7^ Although the proapoptotic genes can be upregulated in response to cytokine or growth factor withdrawal,^8^ the regulatory mechanism is largely unclear.

On the basis on their structures and functions, the members of the Bcl-2 superfamily are divided into prosurvival Bcl-2, proapptotic Bax/Bak, and proapoptotic BH3-only family members.^9^ Among proapoptotic Bcl-2 members, Bim has an important role in initiating the mitochondria-dependent caspase-3 activation that is responsible for endothelial cell death, which is a critical process in the pathogenesis of retinopathy.^10^ In contrast, the antiapoptotic Bcl-2 proteins inhibit the intrinsic apoptotic pathway and also limit the caspase-1 activation that is involved in inflammation and pyroptosis^11^ by preventing the assembly of the NOD-like receptor containing pyrin domain 3 (NLRP3) inflammasome.^12, 13^ Therefore, the balance between antiapoptotic and proapoptotic Bcl-2 proteins is an important determinant in regulating endothelial dysfunction and apoptosis.

MicroRNAs (miRNAs) post-transcriptionally inhibit the expression of specific genes by binding to the 3′-untranslated region (3′-UTR) of target mRNAs.^14^ MiRNA plays an important role in the pathogenesis of cardiovascular diasease.^15^ A number of miRNAs have been shown to induce endothelial cell dysfunction or mitochondria-mediated apoptosis, contributing to cardiovascular diseases,^16^ In fact, several miRNAs, including miR-483-3p, miR-221, miR-222, and miR-17-5p, are overexpressed in tumor cells and contribute to antiapoptosis and drug resistance via downregulation of the proapoptotic Bcl-2 family members Puma, Bim, and Noxa.^8, 17, 18, 19^ However, the involvement of miRNAs in the intrinsic apoptosis pathway that responds to cellular stresses by regulating proapoptotic Bcl-2 gene expression is largely unknown.

In this study, we found that miRNA-101-3p (miR-101-3p) was downregulated in response to serum deprivation and induced endothelial cell apoptosis by targeting the 3′-UTR of Bim mRNA. These findings suggest that miR-101-3p plays an important role in endothelial cell dysfunction and may be a therapeutic target for treating cardiovascular disorders.

## Results

### Serum withdrawal decreases miRNA-101-3p biogenesis

**Figure 1:**
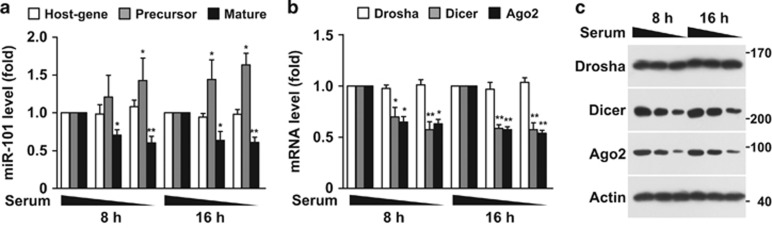
Serum deprivation decreases miR-101-3p biogenesis by downregulating Dicer and Ago2 expression. (**a**) HUVECs were cultured in 5, 1, and 0 FBS-supplemented M199 for 8 and 16 h. Levels of RCL1 and precursor and mature miR-101 s were determined by qRT-PCR. (**b** and **c**) Under the same experimental conditions, Drosha, Dicer, and Ago2 mRNA and protein levels were determined by qRT-PCR and Western blot analysis. *P<0.05 and **P<0.01 *versus* 5% serum

Because our previous study showed that hypoxia-responsive miR-101 regulates endothelial cell function related to angiogenesis and survival,^20^ we initially examined the effect of serum deprivation on miR-101-3p biogenesis in human umbilical vein endothelial cells (HUVECs). When cultured HUVECs with different serum concentrations (5, 1, and 0%) for 8 and 16 h, the precursor miR-101-3p level was inversely proportional to serum concentration, without changing the expression of its host gene RNA 3′-terminal phosphate cyclase-like 1 (RCL1); however, the level of mature miR-101-3p decreased significantly (Figure 1a), and similar results were also observed in several tumor cell lines (Supplementary Figure S1a). These data suggest that serum depletion inhibits miR-101-3p biogenesis at the post-transcriptional level. Because Drosha, Dicer, and Argonaute 2 (Ago2) are crucial for post-transcriptional miRNA biogenesis,^21^ we determined these gene expression levels in serum-deprived HUVECs. Serum deprivation diminished the mRNA and protein levels of Dicer and Ago2, but not Drosha (Figures 1b and c). These data suggest that serum deprivation inhibits miR-101-3p biogenesis, without changing RCL1 expression, by decreasing Dicer and Ago2 expression. Hawkins *et al.* proposed using next-generation sequencing that Dicer deletion can downregulate miR-181c expression, although the result was not reproducible by quantitative real-time PCR (qRT-PCR) analysis.^22^ However, our results showed that miRNA-181c level was unchanged in serum-deprived cells (Supplementary Figure S1b).

### Serum deprivation elicits apoptosis by decreasing miRNA-101-3p biogenesis

**Figure 2:**
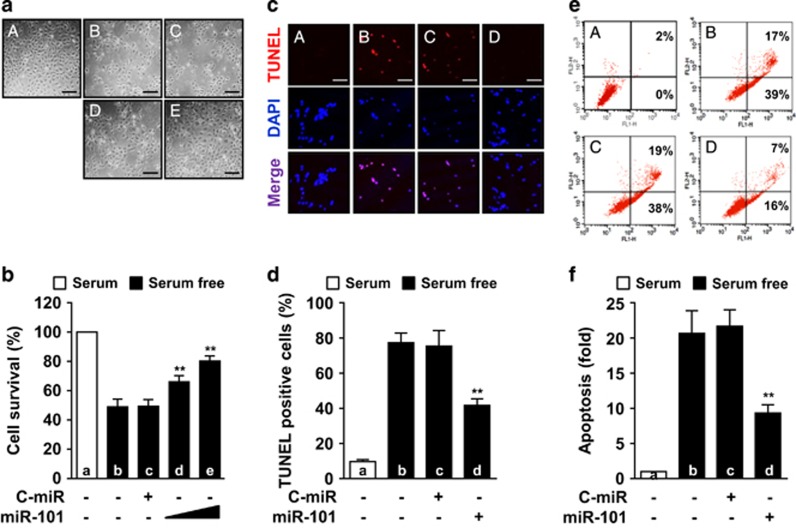
MiR-101-3p prevents serum deprivation-induced apoptosis. HUVECs were transfected with control miRNA (C-miR) or miRNA-101-3p mimic (miR-101) and cultured in serum-free or 5% FBS-supplemented media for 24 h (TUNEL and FACS analysis) or 30 h (cell viability). (**a** and **b**) Cell viability was evaluated by microscopy (**a**) and MTT assay (**b**). Scale bars, 50 *μ*m. A: 5% FBS, B: serum-free, C: serum-free+100 nM C-miR, D: serum-free+50 nM miR-101, and E: serum-free+100 nM miR-101. (**c**) Apoptosis was determined by TUNEL assay, and nuclei were also stained with DAPI. Scale bars, 50 *μ*m. A: 5% FBS, B: serum-free, C: serum-free+C-miR, D: serum-free+100 nM miR-101. (**d**) Quantitation of TUNEL-positive cells. (**e**) Cells were stained with Annexin V and PI and analyzed by FACS. A: 5% FBS, B: serum-free, C: serum-free+100 nM C-miR, D: serum-free+100 nM miR-101. (**f**) Quantitation of cells stained with both Annexin V and PI. *P<0.05 and **P<0.01 *versus* cells transfected with C-miR in serum-free media

Serum withdrawal decreased HUVEC survival with ~50% cell death at 30 h, compared with control cells incubated with 5% fetal bovine serum (FBS). The cell death was prevented in a dose-dependent manner by transfection with 50–100 nM miR-101-3p (Figures 2a and b), suggesting that miR-101-3p plays an important role in regulating serum deprivation-induced cell death. We next performed TUNEL assay to discriminate between apoptosis and other types of cell death. Serum deprivation resulted in a significant increase in the number of TUNEL-positive apoptotic cells compared with control cells, whose apoptotic effect was inhibited by miR-101-3p (Figures 2c and d). Fluorescence-activated cell sorting (FACS) analysis using Annexin V-FITC/PI staining also confirmed that serum deprivation-induced apoptosis, which was inhibited by miR-101-3p (Figures 2e and f). In addition, knockdown of Dicer and Ago2 decreased the miR-101-3p level and promoted endothelial cell apoptosis under serum-free or FBS-supplemented conditions, and this apoptotic cell death was inhibited by miR-101-3p (Supplementary Figures S2a−e and S3a−e). This result suggests that serum deprivation induces endothelial cell apoptosis by suppressing miR-101-3p biogenesis via downregulation of Dicer and Ago2.

### MiR-101-3p downregulated by serum deprivation triggers the intrinsic apoptosis pathway

**Figure 3:**
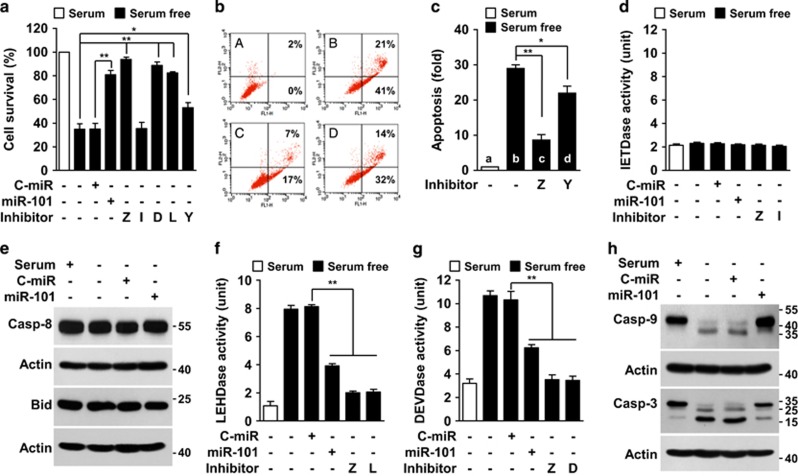
MiR-101-3p inhibits serum deprivation-induced caspase activation and apoptosis. Cells were transfected with C-miR or miR-101-3p and cultured with or without 50 *μ*M of Z-VAD-fmk (Z), Ac-IETD-cho (I), Ac-DEVD-cho (D), Ac-LEHD-cho (L), or Ac-YVAD-cho (Y) in serum-free or 5% FBS-supplemented media for 24 h (FACS and caspase assay) or 30 h (MTT assay). (**a**) Cell viability was measured by MTT assay. (**b** and **c**) Apoptosis was determined serum-deprived HUVECs by FACS analysis. A: 5% FBS, B: serum-free, C: serum-free+Z-VAD-fmk, D: serum-free+Ac-YVAD-cho. (**d**, **f**, and **g**) IETDase, LEHDase, and DEVDase activity were determined in cell lysates by colorimetric assay. (**e**) Caspase-8 and Bid cleavage were determined in cell lysates by western blot analysis. (**h**) Caspases-9/3 activation was determined by Western blot analysis. *P<0.05 and **P<0.01

We examined the role of miR-101-3p in serum deprivation-induced caspase activation. Endothelial cell death in serum-free condition was inhibited by the inhibitors of pan-caspase (z-VAD-fmk), caspase-7/3 (Ac-DEVD-cho), caspase-9 (Ac-LEHD-cho) or caspase-1 (Ac-YVAD-cho), but not by the caspase-8 inhibitor (Ac-IETD-cho) (Figure 3a). Moreover, the cell death was also abolished by transfection with miR-101-3p (Figure 3a). Similarly, z-VAD-fmk and Ac-YVAD-cho also inhibited serum deprivation-induced endothelial cell apoptosis (Figures 3b and c). These results suggest that miR-101-3p downregulated by serum deprivation causes caspase-8-independent apoptosis. As expected, serum-deprived cells did not increase caspase-8-like protease (IETDase) activity, proteolytic caspase-8 activation, and its biological substrate Bid cleavage, and these events were not affected by miR-101-3p (Figures 3d and e). Furthermore, miR-101-3p overexpression inhibited the serum withdrawal-induced increases in caspase-9 (LEHDase)-like and caspase-7/3 (DEVDase)-like activities, as did z-VAD-fmk, Ac-LEHD-cho, and Ac-DEVD-cho (Figures 3f and g). Similarly, miR-101-3p inhibited proteolytic caspases-9/3 activation in serum-deprived cells (Figure 3h). In contrast, knockdown of Dicer or Ago2, an upstream mediator of miR-101-3p biogenesis, increased caspase-9-like (LEHDase), caspase-7/3-like (DEVDase), and caspase-1-like (YVADase) activities independent of serum status, and these enzyme activities were significantly reduced by miR-101-3p (Supplementary Figures S2f and S3f). These data suggest that downregulated miR-101-3p plays an important role in triggering the caspase-8-independent or intrinsic apoptosis pathway that is evoked by serum deprivation.

### Serum deprivation increases Bim expression via decreased miR-101-3p biogenesis

**Figure 4:**
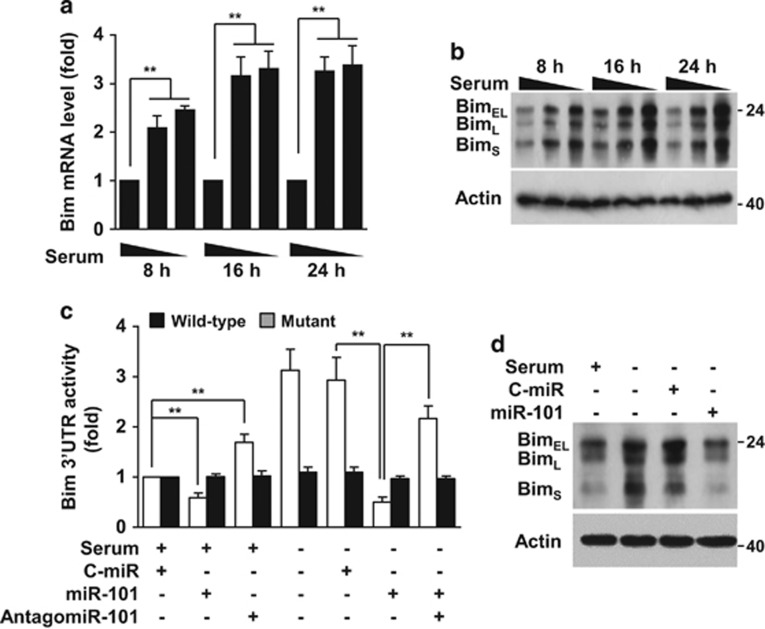
Serum deprivation increases the expression of Bim, a target of miR-101-3p. Cells were cultured in 5, 1, and 0% FBS-supplemented media in the presence of 50 *μ*M Z-VAD-fmk for 16 h. (**a** and **b**) Bim mRNA and protein levels were determined at different time points by qRT-PCR and western blot analysis. (**c** and **d**) Cells transfected with C-miR, miRNA-101, antagomiR-101-3p or psiCHECK-2/Bim 3′-UTR (wild-type or mutant) were cultured in 5% FBS-supplemented or serum-free media containing 50 *μ*M Z-VAD-fmk for 16 h. (**c**) Bim 3′-UTR activity was determined using a dual-luciferase reporter assay system. (**d**) Bim protein level was determined by western blot analysis. Bim_EL_, Bim_L_, and Bim_S_ indicate extra long, long, and short forms of Bim. **P<0.01

Because serum or trophic factor deprivation induces endothelial cell apoptosis,^23^ we predicted the highly reliable apoptosis-related genes of miR-101-3p using TargetScan, microRNA.org, and miRDB. As a result, we selected Bim as a potential target gene of miR-101-3p given that its target site is conserved among mammals and three Bim isoforms including extra-long, long, and short forms (Supplementary Figure S4a−c). We further verified Bim as a bona fide target of miR-101-3p through the significant increases in Bim mRNA and protein levels as a result of serum deprivation (Figures 4a and b). In addition, serum-deprived cells increased Bim mRNA 3′-UTR but not its mutant activity, and this increase was suppressed by transfection of miR-101-3p, whose suppressive effect was recovered by antagomiR-101-3p (Figure 4c). Similar results were also observed in serum-supplemented cells transfected with miR-101-3p or anatogomiR-101-3p (Figure 4c). As expected, elevated Bim protein levels in the serum-deprived cells were reduced to the control levels by miR-101-3p overexpression (Figure 4d). In addition, Dicer or Ago2 knockdown increased Bim mRNA levels in HUVECs independent of serum status (Supplementary Figures S2g and S3g). We also confirmed that treatment with anatagomiR-101-3p increased Bim expression and apoptotic cell death in serum-supplemented cells (Supplementary Figure S5a−c). These results suggest that serum deprivation increases Bim expression via downregulation of miR-101-3p, which directly targets the 3′-UTR of Bim mRNA.

### The serum deprivation-induced intrinsic apoptosis pathway is mediated by the miR-101-3p/Bim axis

**Figure 5:**
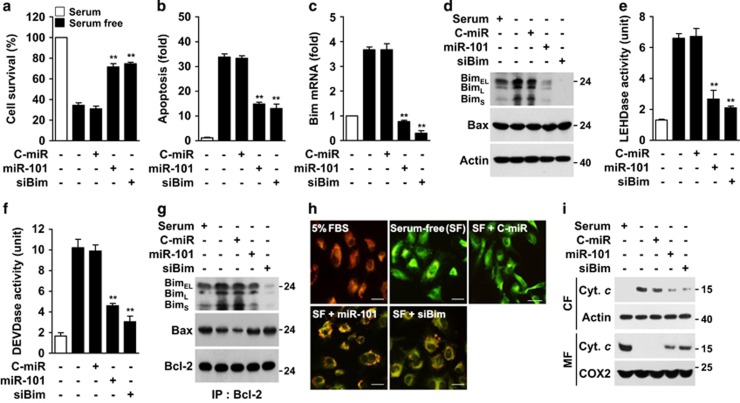
MiR-101-3p regulates serum deprivation-induced intrinsic apoptosis by inhibiting Bim expression. Cells were transfected with C-miR, miR-101 or Bim siRNA (siBim), followed by culture in serum-free or 5% FBS-supplemented media for 12 h (IP and MMP), 16 h (gene and protein expression), 24 h (FACS and caspase assay) or 30 h (cell viability). (**a**) Cell viability was determined by MTT assay. (**b**) Apoptosis was evaluated by FACS analysis after staining with Annexin V and PI. (**c**) Bim mRNA level was determined by qRT-PCR. (**d**) Bim and Bax protein levels were determined by western blotting. (**e** and **f**) LEHDase and DEVDase activity were determined in cell lysates by colourimetric assay. (**g**) Cell lysates were immunoprecipitated with an antibody for Bcl-2. Interaction of Bcl-2 and Bim or Bax was determined by western blotting. (**h**) MMP was analyzed by confocal microscopy using a JC-1 dye. Scale bars, 20 *μ*m. (**i**) Cytochrome *c* levels were determined in the cytosolic (CF) and mitochondrial fractions (MF) by Western blot analysis. COX2; Cytochrome *c* oxidase subunit II. **P<0.01 *versus* cells transfected with C-miR in serum-free media

Bim, a proapoptotic BH3-only member, activates the mitochondrial pathway of apoptosis by interacting with Bcl-2.^24^ We examined whether the miR-101-3p/Bim axis would be involved in apoptosis induced by serum deprivation. Apoptotic cell death of serum-deprived endothelial cells was significantly inhibited by transfection with miR-101-3p or Bim siRNA (Figures 5a and b). Both transfections effectively blocked the serum deprivation-induced increases in Bim mRNA and protein levels, but not the expression of Bax, an internal control gene that is not targeted by miR-101-3p (Figures 5c and d). Moreover, miR-101-3p overexpression and Bim knockdown significantly inhibited the serum deprivation-mediated increases in caspases-9/3-like protease activity (Figures 5e and f). Because Bim is a trigger of the intrinsic apoptosis pathway,^5^ we examined the role of miR-101-3p in the mitochondrial events of apoptosis. Serum withdrawal increased the formation of the Bcl-2/Bim complex through upregulation of Bim expression, resulting in a significant reduction of Bcl-2/Bax interaction, and these events were inhibited by transfection with miR-101-3p or Bim siRNA (Figure 5g). Furthermore, miR-101-3p overexpression or Bim knockdown prevented the loss of mitochondrial membrane potential (MMP) and cytochrome *c* release from mitochondria in serum-deprived cells (Figures 5h and i). These data suggest that serum deprivation triggers the intrinsic apoptosis pathway via the miR-101-3p/Bim axis responsible for cytochrome *c* release from mitochondria.

### Serum deprivation-induced miR-101-3p/Bim axis causes mitochondria-dependent caspase-1 activation

**Figure 6:**
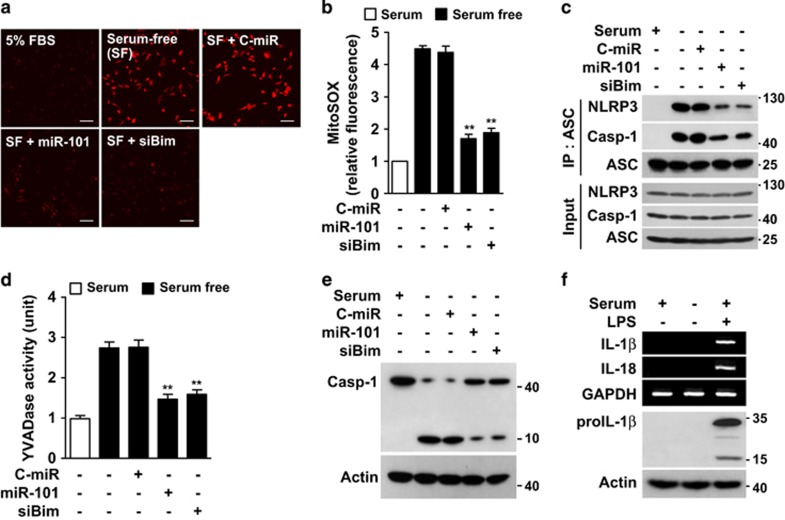
MiR-101-3p blocks serum deprivation-induced mitochondrial ROS production and caspase-1 activation. Cells were transfected with C-miR, miR-101 or siBim, followed by culture in serum-free or 5% FBS-supplemented media for 12 h (ROS assay and IP) or 24 h (caspase assay). (**a**) Mitochondrial ROS generation was identified by confocal microscopy using MitoSOX. Scale bars, 50 *μ*m. (**b**) Relative fluorescence intensity was quantitated by Image J software. (**c**) Cell lysates were immunoprecipitated with an antibody for ASC. Interaction among NLRP3, caspase-1, and ASC was determined by Western blot analysis. (**d**) YVADase activity was determined in cell lysates by colorimetric assay. (**e**) Proteolytic caspase-1 activation was determined by Western blotting. (**f**) Cells were cultured in serum-free or 5% FBS-supplemented media for 16 h. Levels of IL-1*β* and IL-18 were determined by RT-PCR and Western blotting. LPS (100 ng/ml) was used as a positive control. **P<0.01 *versus* cells transfected with C-miR in serum-free media

Mitochondria-derived reactive oxygen species (ROS) stimulate the formation of the NLRP3 inflammasome,^12^ leading to caspase-1 activation that induces apoptosis.^25^ We found in this study that serum deprivation significantly increased mitochondrial ROS production, which was inhibited by transfection with miR-101-3p or Bim siRNA (Figures 6a and b). Serum withdrawal stimulated inflammasome assembly through the interaction of a caspase activation and recruitment domain (ASC) with NLRP3 and caspase-1, and this complex formation was decreased by transfection with miR-101-3p or Bim siRNA (Figure 6c). Under the same experimental condition, the serum deprivation-induced increases in YVADase activity and proteolytic caspase-1 activation were suppressed by miRNA-101-3p or Bim siRNA (Figures 6d and e). Since caspase-1 cleaves proIL-1*β* and proIL-18 to their mature forms, we next examined whether serum deprivation would regulate expression and maturation of these cytokines. Serum deprivation did not increase detectable levels of IL-1*β* and IL-18 expression or IL-1*β* maturation (Figure 6f). These data suggest that the miR-101-3p/Bim axis promotes mitochondrial ROS generation, which stimulates inflammasome assembly and caspase-1 activation, but not IL-1*β* and IL-18 expression and maturation, in serum-deprived endothelial cells.

### Antioxidants inhibits serum deprivation induces caspase-1-mediated apoptosis

**Figure 7:**
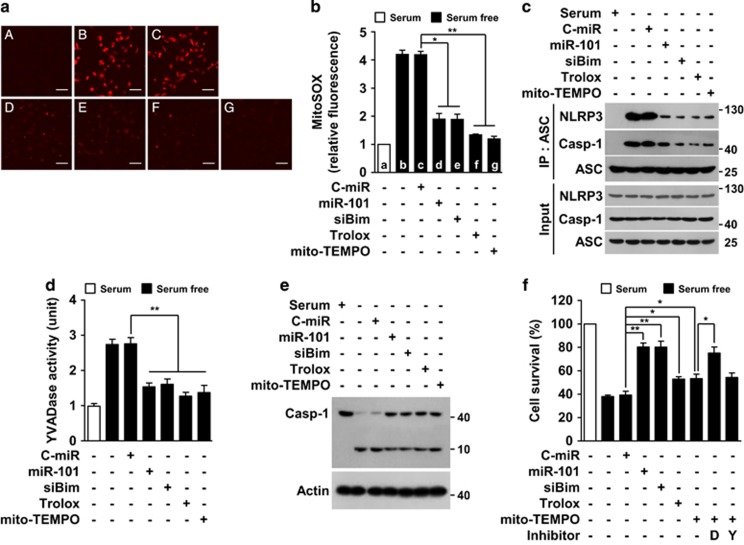
MiR-101-3p blocks serum deprivation-induced mitochondrial ROS production, NLRP3 inflammasome activation, and caspase-1 activation. Cells were transfected with C-miR, miR-101 or siBim, followed by treatment with Trolox, Mito-TEMPO, Ac-DEVD-cho (D) or Ac-YVAD-cho (Y) in serum-free media for 12 h (ROS assay and IP), 24 h (caspase assay) or 30 h (MTT assay). (**a**) Mitochondrial ROS generation was identified by MitoSOX-based confocal microscopy. Scale bars, 50 *μ*m. A: 5% FBS, B: serum-free, C: serum-free+100 nM C-miR, D: serum-free+100 nM miR-101, E: serum-free+100 nM siBim, f: serum-free+10 *μ*M Trolox, g: serum-free+10 *μ*M mito-TEMPO. (**b**) Fluorescence intensity was determined by Image J software. (**c**) NLRP3 inflammasome activation was determined by Western blotting following IP. YVADase activity (**d**) and proteolytic caspase-1 activation (**e**) were determined in cell lysates by colourimetric assay and Western blot analysis. (**f**) Cell viability was also determined by MTT assay. *P<0.05 and **P<0.01

We examined using antioxidants whether mitochondrial ROS production is an important factor for serum deprivation-induced caspase-1 activation and apoptosis. As shown by transfection with miR-101-3p or Bim siRNA (Figures 6a and b), treatment with Trolox (antioxidant) or Mito-TEMPO (mitochondria-targeted antioxidant) also effectively inhibited mitochondrial ROS generation in serum-deprived HUVECs (Figures 7a and b). Similarly, both antioxidants suppressed serum starvation-induced assembly of the NLRP3 inflammation, which consists of NLRP3, ASC, and caspase-1 (Figure 7c). Moreover, the antioxidants significantly inhibited the serum-deprived increases in YVADase activity and proteolytic caspase-1 activation (Figures 7d and e). These results suggest that ROS generated from mitochondria by the miR-101-3p/Bim pathway stimulates inflammasome-mediated caspase-1 activation. Further examination of whether mitochondrial ROS is involved in endothelial cell apoptosis via caspase-1 activation showed that the antioxidants Trolox and Mito-TEMPO protected cells from serum deprivation-induced apoptosis, and the protective effect of Mito-TEMPO was further increased by co-treatment with Ac-DEVD-Cho, but not with Ac-YVAD-Cho (Figure 7f), suggesting that caspase-1 activation by ROS-mediated formation of inflammasome promotes apoptosis via activation of DEVDase. Thus, the miR-101-3p/Bim axis plays a key role in serum deprivation-induced apoptosis by promoting inflammasome assembly and caspase-1 activation via mitochondrial ROS production. However, the mitochondrial ROS production was not significantly inhibited by caspase inhibitors, Z-VAD-fmk, Ac-YVAD-cho, Ac-LEHD-cho or Ac-DEVD-cho (Supplementary Figure S6a−c), indicating that caspases are not involved in serum deprivation-induced ROS generation.

### Inflammasome-mediated caspase-1 activity induces apoptosis by activating caspase-7, but not caspase-3

**Figure 8:**
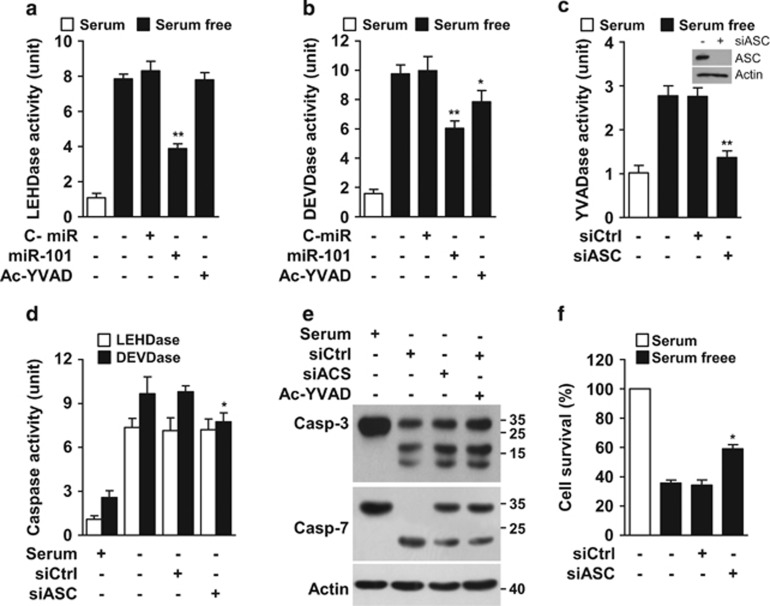
Caspase-1 induces serum deprivation-induced apoptosis by increasing caspase-7 activation. Cells were transfected with C-miR, miR-101, control siRNA (siCtrl) or ASC siRNA (siASC), followed by treatment with Ac-YVAD-cho in serum-free media for 24 h (caspase assay) or 30 h (cell viability). (**a**−**d**) Caspase activity was determined in cell lysates by colorimetric assay. (**e**) Caspases-3/7 activation was determined by Western blot analysis. (**f**) Cell viability was evaluated by MTT assay. *P<0.05 and **P<0.01 *versus* cells transfected with C-miR or siCtrl in serum-free media

Since caspase-1 induces pyroptosis by activating executive caspases, like DEVDase,^26^ we examined whether caspase-1 would activate other caspases in serum-deprived HUVECs. LEHDase activity increased by serum starvation was inhibited by miR-101-3p, but not Ac-YVAD-cho (Figure 8a), whereas DEVDase activity was partially inhibited by both miR-101-3p and Ac-YVAD-cho (Figure 8b), suggesting that caspase-1 stimulates DEVDase activity. We next examined the effect of ASC (an essential component of inflammasome complex) on caspase-7/3-like (DEVDase) activity and cell death using ASC siRNA. ASC knockdown inhibited YVADase and DEVDase activity, but not that of LEHDase, in serum-deprived cells (Figures 8c and d). Because the executioner caspase-7/3 cleave the same substrate Ac-DEVD-pNA used in this study,^27^ we further determined which caspases could be activated by caspase-1. Both caspases were activated by serum starvation, whereas only caspase-7 activation was partially inhibited by ASC siRNA and Ac-YVAD-cho (Figure 8e). As expected, ASC knockdown protected the cells from serum deprivation-induced cell death (Figure 8f). These results suggest that inflammasome-mediated caspase-1 activity induces pyroptosis by activating caspase-7, but not caspase-3, in serum-deprived endothelial cells.

## Discussion

MiRNA synthesis is a two-step sequential process following RNA pol II/III-dependent transcription of pri-miRNA, with both nuclear and cytoplasmic cleavages to pre-miRNA and mature miRNA by Drosha and Dicer, respectively.^21^ In fact, serum deprivation has been shown to induce endothelial cell apoptosis via caspase-3 activation by decreasing Dicer expression, and these unfavorable phenomena were abrogated by Dicer overexpression,^28^ indicating that miRNAs are critically involved in serum deprivation-induced apoptosis. Moreover, genetic deletion of Dicer upregulated the proapoptotic target gene Bim, reflecting apoptotic cell death.^22^ These data suggest that serum deprivation promotes apoptosis by decreasing Dicer expression, probably due to decreased biogenesis of miRNAs that target apoptosis-associated genes. Here we found that serum withdrawal decreased Dicer expression, thereby leading to increased susceptibility to apoptosis through downregulation of miR-101-3p, which targets the 3′ UTR of Bim mRNA.

The mature miRNA duplex generated by Dicer is loaded into the RNA-induced silencing complex (RISC, consisting of Dicer, TRBP, and Ago2) and generates a single-stranded mature miRNA by cleaving the passenger strand. Activated RISC binds to the target mRNA through complementary base-pairing between the guide strand and the 3′-UTR of the target, resulting in the cleavage of target mRNA and post-transcriptional gene silencing.^29^ Among the components of RISC, Ago2 is critically involved in silencing the target gene. Ago2 is downregulated in serum-deprived HeLa cells, although an exact mechanism has not been elucidated.^30^ Similarly, we found that serum withdrawal suppressed Ago2 expression in HUVECs and decreased the expression of the miR-101-3p target gene Bim. Our data also showed that either Dicer or Ago2 knockdown induced apoptosis via increased Bim expression by inhibiting miR-101-3p biogenesis. That is, serum starvation upregulates Bim expression via miR-101-3p downregulation by decreasing Dicer and Ago2 expression.

It was shown that miR-101 can sensitize tumor cells chemotherapeutic drug-induced apoptosis by indirectly upregulating Bim expression in an EZH2-dependent epigenetic control manner.^27^ However, we demonstrated that miR-101-3p directly targets the 3′-UTR of Bim mRNA, resulting in increased Bim expression in serum-deprived endothelial cells. It is also possible that miR-101 regulates expression of cell survival genes, such as Mcl-1 and AMPK, by targeting their 3′-UTRs.^31, 32^ These target genes have the same canonical seed-matched sequences of 8mer (Supplementary Figure S7a), leading to similar responses to miR-101-3p overexpression (Supplementary Figure S7b and c). However, Bim expression was higher than ther other genes in serum-deprived cells (Supplementary Figure S7d−f). This phenomenon may have occurred via the cooperative action of miR-101-3p with other miRNAs or factors that are responded by serum deprivation or Dicer deletion.^33, 34, 35, 36, 37^ Therefore, further study is needed to understand the interaction between miR-101-3p and Bim expression in serum-derived endothelial cells.

In contrast to the antiapoptotic function of decreased miR-101-3p level in tumor cells,^31, 38^ its downregulation promoted apoptosis in mouse primary cardiac fibroblasts exposed to hypoxia.^39^ Similarly, miR-101 is downregulated in Dicer-knockout mice and increased apoptosis via elevation of Bim expression.^22^ Consistent with this finding, our data showed that serum deprivation-induced endothelial apoptosis through downregulation of miR-101-3p, which targets Bim. All of these evidences suggests that miR-101-3p has a distinct cellular function, such as tumor-suppressive (e.g., apoptotic) *versus* antiapoptotic (survival) activity in tumor cells and normal cardiovascular cells, respectively. There are two possible explanations for this phenomenon. First, miR-101-3p may differently regulate the target gene expression in tumor and normal cells, as shown in a distinct gene expression pattern at the transcriptional level in a cell type (or cell differentiation stage)-specific manner. Another explanation may be related to the characteristics of the miRNA-target gene network that a miRNA targets multiple genes in a sequence-specific, but not gene-specific manner.

Apoptosis is mediated by two central pathways, an extrinsic pathway involving cell surface receptors and an intrinsic pathway using mitochondria.^40^ Activation of death receptors by their respective ligands induces sequential intracellular signal events, such as caspase-8 activation, Bid cleavage, mitochondrial cytochrome *c* release, and caspases-9 activation, resulting in proteolytic cleavage of the executioner caspase-7/3 and subsequent induction of apoptosis. On the other hand, intrinsic death stimuli induce cytochrome *c*-mediated apoptotic signal cascades, without caspase-8 activation.^41^ We observed that serum-deprived HUVECs induced increased mitochondrial cytochrome *c* release and caspases-9/7/3 activation, without caspase-8-mediated cleavage of Bid, suggesting that serum deprivation induces endothelial cell apoptosis via the intrinsic pathway.^42^ Moreover, endothelial cell apoptosis induced by serum deprivation was effectively inhibited by a miR-101-3p mimic, but not by a caspase-8 inhibitor. These data indicate that miR-101-3p plays an important mediator role in mitochondria-dependent intrinsic apoptosis in serum-deprived endothelial cells.

The intrinsic apoptosis pathway is usually regulated by a balance between proapoptotic and prosurvival Bcl-2 family proteins proteins. Among proapoptotic Bcl-2 proteins, Bim interacts with prosurvival Bcl-2 members including Bcl-2 and Bcl-xL to allow the release of the BH3-only proapoptotic Bax and Bak proteins from their heterodimeric complexes.^24^ The release of these proteins in turn drives the apoptogenic cytochrome *c* release from the mitochondria, resulting in the activation of caspase-7/3 that execute apoptosis. Alternatively, Bim can directly activate Bax or Bak through interaction with them; thus, Bim is an important component for initiating the intrinsic apoptosis pathway. In accordance with a previous study,^43^ we found that Bim plays a central role in apoptosis in response to serum deprivation. Taken in combination with recent findings, it would appear that Bim is an important mediator of mitochondria-dependent endothelial cell apoptosis induced by serum or growth factor deprivation.^25, 43^ However, the mechanism linked to how Bim expression is regulated in response to serum starvation has not been clearly defined. A recent study showed that serum withdrawal induced endothelial apoptosis by downregulating miR-17-5p, which increases Bim expression by targeting its 3′-UTR.^33, 36^ In addition, Dicer ablation increases cell death by downregulating miR-20 and miR-302, which inhibit Bim expression by targeting its 3′-UTR,^37^ indicating that Bim is upregulated in a miRNA-dependent manner. It suggests that upregulation of Bim in serum-deprived endothelial cells can be cooperatively induced by several miRNAs, including miR-101-3p.

Dicer is downregulated in HUVECs and fibroblasts by serum starvation, and this effect is abolished by angiogenic stimuli, such as vascular endothelial cell growth factor (VEGF) or sphingosine-1-phosphate (S1P), but not basic fibroblast growth factor and lysophosphatidic aid.^28^ This suggests that VEGF and S1P are important serum components for maintaining Dicer expression and preventing apoptosis. Indeed, inhibiting VEGF activity induces endothelial cell apoptosis by increasing Bim expression and vice versa.^44^ In this study, we demonstrated that serum deprivation increased Bim expression via the downregulated biogenesis of miR-101-3p that targets the 3′-UTR of Bim mRNA, resulting in the increases in Bax activity and mitochondria-dependent apoptosis. In addition, miR-101-3p biogenesis was also found to be decreased by downregulation of Dicer in serum-deprived HUVECs, although the regulatory mechanism has not been clarified as yet.

**Figure 9:**
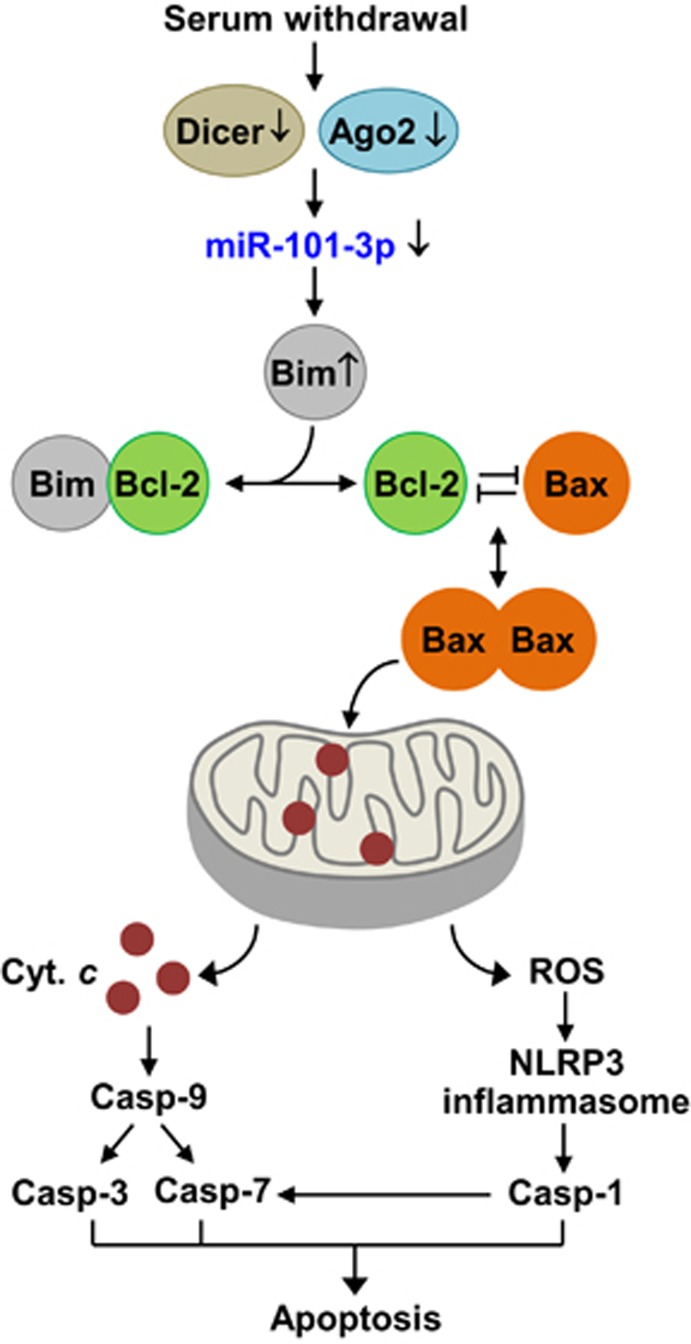
A schematic diagram showing the functional involvement of miR-101-3p in serum withdrawal-induced apoptosis by targeting Bim. Serum deprivation inhibits miR-101-3p biogenesis by downregulating Dicer and Ago2 expression, leading to increased Bim expression. Bim interacts with Bcl-2 to allow the release of Bax, which drives cytochrome *c* release and ROS generation from mitochondria. Both events induce intrinsic apoptosis via caspases-9/7/3 activation and inflammasome-mediated caspase-1/7 activation, respectively

Although caspase-3 is a major executive effector of apoptosis, accumulating evidence recognizes caspase-1 as another contributor to non-canonical apoptosis induced by homocysteine, inflammation, and serum deprivation.^11, 26, 45^ Caspase-1 is generally activated by the formation of the NLRP3 inflammasome complex consisting of NLRP3, ASC, and procaspase-1. Although the NLRP3 inflammasome complex is essential for caspase-1-mediated maturation of IL-1*β* and IL-18,^46^ this complex also mediates pyroptosis via caspase-1 activation.^15^ Indeed, caspase-1 activation through inflammasome formation plays an important role in homocysteine-induced endothelial dysfunction by inducing pyroptosis and apoptosis,^11^ likely by activating caspase-7, but not caspase-3.^26^ Similarly, our results showed that caspase-1 activated caspase-7, but not caspase-3, in serum-deprived endothelial cells. NLRP3 inflammasome activity is positively regulated by ROS derived from mitochondria,^12^ and the ROS production can be elevated by Bim-dependent mitochondrial dysfunction.^47^ We found that decreased miR-101-3p in serum-deprived cells promoted mitochondrial ROS-mediated caspase-1 activation and apoptosis by upregulating Bim expression. However, serum deprivation had no effect on expression and maturation of IL-1*β* and IL-18, indicating that these cytokines are not involved in serum derivation-induced apoptosis. These results suggest that serum deprivation-induced apoptosis can be mediated by the miR-101-3p/Bim axis, which stimulates two distinct pathways, cytochrome *c*-mediated caspase-7/3 activation and ROS-dependent caspase-1/7 activation (Figure 9). Therefore, antioxidants may prevent apoptosis mediated by caspase-1/7, but not caspase-3.

Taken together, our results indicate that miR-101-3p downregulation is a key step in endothelial dysfunction induced by serum or growth factor withdrawal. We also provide evidence that miR-101-3p is an important determinant of endothelial function and vascular homeostasis in pathological conditions, such as trophic factor depletion, implicating miR-101-3p as a therapeutic target in cardiovascular disease, including atherosclerosis.

## Materials and methods

### Materials

Cell culture media supplements and Lipofectamine RNAiMAX were purchased from Invitrogen Life Technologies (Invitrogen, Carlsbad, CA, USA). MitoSOX was purchased from Molecular Probes (Eugene, OR, USA). Antibodies against human Drosha, Dicer, Ago2, caspases-1/-3/-9, Bim, Bcl-2, COX2, NLRP3, ASC, IL-1*β*, Mcl-1 and siRNAs against Bim, Dicer, Ago2, and ASC were purchased from Santa Cruz Biotechnology (Santa Cruz, CA, USA). Antibodies against human caspase-8, Bid, Bax, and cytochrome *c* were purchased from BD Biosciences (San Jose, CA, USA). Antibody against human AMPK*α* was purchased from Cell Signaling Technology (Beverly, MA, USA). MiRNeasy Mini kit, miR-101-3p mimic, miScript SYBR Green PCR kit, and real-time PCR primers were purchased from Qiagen (Hilden, Germany). Caspase inhibitors and substrates were purchased from Alexis Corporation (San Diego, CA, USA). Other chemicals were purchased from Sigma (St. Louis, MO, USA).

### Cell culture and treatment

HUVECs were cultured as previously described.^20^ Cells were transfected with 100 nM of control miR, miR-101-3p mimic, antagomiR-101-3p or siRNAs (100 nM) targeted to Bim, Dicer, Ago2, ASC using Lipofectamine RNAiMAX for 24 h and cultured with or without caspase inhibitors (50 *μ*M), Trolox (10 *μ*M) or Mito-TEMPO (10 *μ*M) in M199 supplemented with 0 or 5% FBS for 8−30 h. The cells were used for further analyses. In addition, tumor cells (MCF-7, HeLa, and HCT116) were also cultured in RPMI medium supplemented with 0% or 5% FBS.

### Polymerase chain reaction

Cells were cultured in serum-free or 5% FBS-supplemented media containing 50 *μ*M z-VAD-fmk for 16 h, and miRNAs were isolated using a miRNeasy mini kit according to manufacturers’ protocols. cDNA for determining miRNAs was synthesized from 1 *μ*g of miRNAs using a miScript II RT kit. Levels of precursor and mature miR-101-3p were determined by qRT-PCR using a miScript SYBR Green PCR kit with miR-101-3p-specific and universal primers (Qiagen) as previously described.^20^ Levels of Drosha, Dicer, Ago2, Bim, Mcl-1, AMPK and cytokine mRNAs were determined by HiPi Real-Time PCR 2x Master Mix (SYBR Green+ROX) (Elpis, Daejeon, Korea) with Rotor-Gene Q (Qiagen) using target-specific primers. The primers used in this study are listed in Supplementary Table S1.

### Western blot analysis

Western blot analysis for target proteins in whole-cell lysates and in cytosolic and mitochondrial fractions was performed as previously described.^20^

### Cell viability assay

Cell viability was evaluated by 3-[4,5-cimethylthiazol-2-yl]-2,5-diphenyl tetrazolium bromide (MTT; Sigma-Aldrich, St. Louis, MO, USA) assay. Cells were transfected with miR-101-3p mimic or Bim siRNA using Lipofectamine RNAiMAX and cultured for 30 h, followed by incubation with MTT solution (1 mg/ml) for 2 h at 37 °C. Cell viability was determined by measuring absorbance at 550 nm using a microplate reader.

### TUNEL assay

Cells were transfected with control miRNA (C-miR) or miR-101-3p mimic and cultured in serum-free or 5% FBS-supplemented media for 24 h. Coverslips with adherent cells were fixed in 4% paraformaldehyde for 15 min at room temperature. DNA fragmentation, which indicates apoptosis, was analyzed according to the manufacturer's instructions using terminal deoxynucleotidyl transferase with peroxide-12-UTP nick-end labeling (TUNEL) (Roche Molecular Biochemicals, Indianapolis, IN, USA). Cells were counterstained with DAPI, mounted cell side down on a microscope slide, and analyzed by confocal microscopy. TUNEL-positive cells appeared red.

### Annexin V-FITC staining

Cells were transfected with C-miR, miR-101-3p mimic or Bim siRNA and cultured in serum-free or 5% FBS-supplemented media for 24 h. Apoptosis was determined using Annexin V-FITC/PI according to the manufacturer’s instructions (BD Biosciences, San Jose, CA, USA). Ten thousand cells were counted for three independent experiments. Apoptosis was evaluated by measuring the total amount of Annexin V^+^/PI^-^ cells and Annexin V^+^/PI^+^ cells.

### Caspase activity assay

Cells were transfected with C-miR or miR-101-3p mimic and cultured with or without 50 *μ*M of Z-VAD-fmk (Z), Ac-IETD-cho (I), Ac-DEVD-cho (D), Ac-LEHD-cho (L), or Ac-YVAD-cho (Y) in serum-free or 5% FBS-supplemented media for 24 h. Caspase activity was determined by measuring proteolytic cleavage of the chromogenic substrates, Ac-IETD-pNA (caspase-8-like activity), Ac-LEHD-pNA (caspase-9-like activity), Ac-DEVD-pNA (caspase-7/3-like activity), or Ac-YVAD-pNA (caspase-1-like activity) as described previously.^47^ One unit of caspase activity is defined as an increase in absorbance of 0.1 at 405 nm per mg protein per hour.

### Luciferase assay

Cells transfected with C-miR or miR-101-3p mimic or psiCHECK-2/Bim 3′-UTR (wild-type or mutant) were cultured in serum-free or 5% FBS-supplemented media containing 50 *μ*M Z-VAD-fmk for 16 h. The 3′UTR (~0.7 kb) of Bim was prepared from human genomic DNA by PCR using the following wild-type primers, 5′-ATTCTCGAGTGTACTCACGTGCCAGTC-3′ (forward) and 5′-TTAGCGGCCGCACTCACAATATATACATT-3′ (reverse). The 3’UTR (~0.7 kb) of Bim was prepared from wild-type by PCR using the following mutant primers, 5′-TGACTGGATGTCTCTGGAATTTATGTATCTGGTTATCA-3′(forward) and 5′-TGATAACCAGATACATAAATTCCAGAGACATCCAGTCA-3′ (reverse). The PCR products were ligated at the *Xho*I and *Not*I sites of psiCHECK-2 vector (Promega, Madison, WI, USA). Reporter activity was assayed using a dual-luciferase report assay kit (Promega).

### Immunoprecipitation

Cells transfected with C-miR, miR-101-3p mimic or Bim siRNA were cultured in serum-free or 5% FBS-supplemented media for 12 h. Cell lysates (2 mg of protein) were incubated with an antibody (1 *μ*g) for Bcl-2 or ASC in RIPA buffer with gentle agitation overnight at 4 °C. The lysates were then incubated with protein G-Sepharose bead slurry (100 *μ*l, Millipore, Merck KGaA, Darmstadt, Germany), and immune complexes were collected by centrifugation. Immunoprecipitates were separated by SDS-gel electrophoresis, followed by western blot analysis using the indicated antibodies.

### Measurement of MMP

Cells transfected with C-miR, miR-101-3p mimic or Bim siRNA were cultured in serum-free or 5% FBS-supplemented media for 12 h. MMP was monitored by confocal microscopy. Cells were stained with 2 *μ*mol/l of JC-1 (Molecular Probes) for 30 min at 37 °C. Fluorescence intensities were determined at the single-cell level by confocal microscopy. Data are expressed as a normalized ratio of the fluorescence intensity of the monomeric form (low MMP, green) to the JC-1-aggregate form (high MMP, red).

### Measurement of mitochondrial ROS production

Mitochondrial ROS levels were measured using the mitochondrial superoxide indicator MitoSOX Red. Cells transfected with C-miR, miR-101-3p mimic or Bim siRNA were cultured in serum-free or 5% FBS-supplemented media for 12 h. Cells were loaded with MitoSOX Red (5 *μ*M) for 10 min. After being washed with PBS, the accumulation of red fluorescence (excitation 510 nm, emission 580 nm) was determined by confocal microscopy.

### Statistical analysis

Quantitative data are expressed as the mean±S.D. of at least three separate experiments. Statistical significance was determined using either one-way ANOVA or unpaired Student’s *t*-test, depending on the number of experimental groups analyzed. Significance was established at *P*<0.05.

## References

[bib1] Hadi HA, Carr CS, Al Suwaidi J. Endothelial dysfunction: cardiovascular risk factors, therapy, and outcome. Vasc Health Risk Manag 2005; 1: 183–198. 17319104 PMC1993955

[bib2] Deanfield JE, Halcox JP, Rabelink TJ. Endothelial function and dysfunction: testing and clinical relevance. Circulation 2007; 115: 1285–1295. 17353456 10.1161/CIRCULATIONAHA.106.652859

[bib3] Kroemer G, El-Deiry WS, Golstein P, Peter ME, Vaux D, Vandenabeele P et al. Classification of cell death: recommendations of the Nomenclature Committee on Cell Death. Cell Death Differ 2005; 2: 1463–1467. 10.1038/sj.cdd.4401724 16247491

[bib4] Galluzzi L, Vitale I, Abrams JM, Alnemri ES, Baehrecke EH, Blagosklonny MV et al. Molecular definitions of cell death subroutines: recommendations of the Nomenclature Committee on Cell Death 2012. Cell Death Differ 2012; 19: 107–120. 21760595 10.1038/cdd.2011.96 PMC3252826

[bib5] de Vries EG, Gietema JA, de Jong S. Tumor necrosis factor-related apoptosis-inducing ligand pathway and its therapeutic implications. Clin Cancer Res 2006; 12: 2390–2393. 16638843 10.1158/1078-0432.CCR-06-0352

[bib6] McClintock DS, Santore MT, Lee VY, Brunelle J, Budinger GR, Zong WX et al. Bcl-2 family members and functional electron transport chain regulate oxygen deprivation-induced cell death. Mol Cell Biol 2002; 22: 94–104. 11739725 10.1128/MCB.22.1.94-104.2002 PMC134234

[bib7] Gross A, McDonnell JM, Korsmeyer SJ. BCL-2 family members and the mitochondria in apoptosis. Genes Dev 1999; 13: 1899–1911. 10444588 10.1101/gad.13.15.1899

[bib8] Delbridge AR, Strasser A. The BCL-2 protein family, BH3-mimetics and cancer therapy. Cell Death Differ 2015; 22: 1071–1080. 25952548 10.1038/cdd.2015.50 PMC4572872

[bib9] Czabotar PE, Lessene G, Strasser A, Adams JM. Control of apoptosis by the BCL-2 protein family: implications for physiology and therapy. Nat Rev Mol Cell Biol 2014; 15: 49–63. 24355989 10.1038/nrm3722

[bib10] Wang S, Park S, Fei P, Sorenson CM. Bim is responsible for the inherent sensitivity of the developing retinal vasculature to hyperoxia. Dev Biol 2011; 349: 296–309. 21047504 10.1016/j.ydbio.2010.10.034 PMC3021136

[bib11] Xi H, Zhang Y, Xu Y, Yang WY, Jiang X, Sha X et al. Caspase-1 inflammasome activation mediates homocysteine-induced Pyrop-apoptosis in endothelial cells. Circ Res 2016; 118: 1525–1539. 27006445 10.1161/CIRCRESAHA.116.308501 PMC4867131

[bib12] Zhou R, Yazdi AS, Menu P, Tschopp J. A role for mitochondria in NLRP3 inflammasome activation. Nature 2011; 469: 221–225. 21124315 10.1038/nature09663

[bib13] Xi H, Zhang Y, Xu Y, Yang WY, Jiang X, Sha X et al. Caspase-1 inflammasome activation mediates homocysteine-induced pyrop apoptosis in endothelial cells. Circ Res 2016; 118: 1525–1539. 27006445 10.1161/CIRCRESAHA.116.308501 PMC4867131

[bib14] Bartel DP. MicroRNAs: target recognition and regulatory functions. Cell 2009; 136: 215–233. 19167326 10.1016/j.cell.2009.01.002 PMC3794896

[bib15] Callis TE, Pandya K, Seok HY, Tang RH, Tatsuguchi M, Huang ZP et al. MicroRNA-208a is a regulator of cardiac hypertrophy and conduction in mice. J Clin Invest 2009; 119: 2772–2786. 19726871 10.1172/JCI36154 PMC2735902

[bib16] Quiat D, Olson EN. MicroRNAs in cardiovascular disease: from pathogenesis to prevention and treatment. J Clin Invest 2013; 123: 11–18. 23281405 10.1172/JCI62876 PMC3533276

[bib17] Veronese A, Lupini L, Consiglio J, Visone R, Ferracin M, Fornari F et al. Oncogenic role of miR-483-3p at the IGF2/483 locus. Cancer Res 2010; 70: 3140–3149. 20388800 10.1158/0008-5472.CAN-09-4456 PMC4303586

[bib18] Zhang CZ, Zhang JX, Zhang AL, Shi ZD, Han L, Jia ZF et al. MiR-221 and miR-222 target PUMA to induce cell survival in glioblastoma. Mol Cancer 2010; 9: 229. 20813046 10.1186/1476-4598-9-229 PMC2939570

[bib19] Li Y, Choi PS, Casey SC, Dill DL, Felsher DW. MYC through miR-17-92 suppresses specific target genes to maintain survival, autonomous proliferation, and a neoplastic state. Cancer Cell 2014; 26: 262–272. 25117713 10.1016/j.ccr.2014.06.014 PMC4191901

[bib20] Kim JH, Lee KS, Lee DK, Kim J, Kwak SN, Ha KS et al. Hypoxia-responsive microRNA-101 promotes angiogenesis via heme oxygenase-1/vascular endothelial growth factor axis by targeting cullin 3. Antioxid Redox Signal 2014; 21: 2469–2482. 24844779 10.1089/ars.2014.5856 PMC4245877

[bib21] Winter J, Jung S, Keller S, Gregory RI, Diederichs S. Many roads to maturity: microRNA biogenesis pathways and their regulation. Nat Cell Biol 2009; 11: 228–234. 19255566 10.1038/ncb0309-228

[bib22] Hawkins SM, Andreu-Vieyra CV, Kim TH, Jeong JW, Hodgson MC, Chen R et al. Dysregulation of uterine signaling pathways in progesterone receptor-Cre knockout of dicer. Mol Endocrinol 2012; 26: 1552–1566. 22798293 10.1210/me.2012-1042 PMC3434527

[bib23] Hogg N, Browning J, Howard T, Winterford C, Fitzpatrick D, Gobé G. Apoptosis in vascular endothelial cells caused by serum deprivation, oxidative stress and transforming growth factor-β. Endothelium 1999; 7: 35–49. 10599559 10.3109/10623329909165310

[bib24] Cotter TG. Apoptosis and cancer: the genesis of a research field. Nat Rev Cancer 2009; 9: 501–507. 19550425 10.1038/nrc2663

[bib25] Sollberger G, Strittmatter GE, Grossi S, Garstkiewicz M, Auf dem Keller U, French LE et al. Caspase-1 activity is required for UVB-induced apoptosis of human keratinocytes. J Invest Dermatol 2015; 135: 1395–1404. 25562666 10.1038/jid.2014.551

[bib26] Lamkanfi M, Kanneganti TD, Van Damme P, Vanden Berghe T, Vanoverberghe I, Vandekerckhove J et al. Targeted peptidecentric proteomics reveals caspase-7 as a substrate of the caspase-1 inflammasomes. Mol Cell Proteomics 2008; 7: 2350–2363. 18667412 10.1074/mcp.M800132-MCP200 PMC2596343

[bib27] Zhang JG, Guo JF, Liu DL, Liu Q, Wang JJ. MicroRNA-101 exerts tumor-suppressive functions in non-small cell lung cancer through directly targeting enhancer of zeste homolog 2. J Thorac Oncol 2011; 6: 671–678. 21270667 10.1097/JTO.0b013e318208eb35

[bib28] Asada S, Takahashi T, Isodono K, Adachi A, Imoto H, Ogata T et al. Downregulation of Dicer expression by serum withdrawal sensitizes human endothelial cells to apoptosis. Am J Physiol Heart Circ Physiol 2008; 295: H2512–H2521. 18978195 10.1152/ajpheart.00233.2008

[bib29] Peterson QP, Goode DR, West DC, Botham RC, Hergenrother PJ. Preparation of the caspase-3/7 substrate Ac-DEVD-pNA by solution-phase peptide synthesis. Nat Protoc 2010; 5: 294–302. 20134429 10.1038/nprot.2009.223 PMC2921128

[bib30] Gibbings D, Mostowy S, Jay F, Schwab Y, Cossart P, Voinnet O. Selective autophagy degrades DICER and AGO2 and regulates miRNA activity. Nat Cell Biol 2012; 14: 1314–1321. 23143396 10.1038/ncb2611 PMC3771578

[bib31] Liu X, Tang H, Chen J, Song C, Yang L, Liu P et al. MicroRNA-101 inhibits cell progression and increases paclitaxel sensitivity by suppressing MCL-1 expression in human triple-negative breast cancer. Oncotarget 2015; 6: 20070–20083. 26036638 10.18632/oncotarget.4039 PMC4652988

[bib32] Liu P, Ye F, Xie X, Li X, Tang H, Li S et al. mir-101-3p is a key regulator of tumor metabolism in triple negative breast cancer targeting AMPK. Oncotarget 2016; 7: 35188–35198. 27145268 10.18632/oncotarget.9072 PMC5085220

[bib33] Koenig MN, Naik E, Rohrbeck L, Herold MJ, Trounson E, Bouillet P et al. Pro apoptotic BIM is an essential initiator of physiological endothelial cell death independent of regulation by FOXO3. Cell Death Differ 2014; 21: 1687–1695. 24971484 10.1038/cdd.2014.90 PMC4211375

[bib34] Kong PJ, Kil MO, Lee H, Kim SS, Johnson GV, Chun W. Increased expression of Bim contributes to the potentiation of serum deprivation-induced apoptotic cell death in Huntington's disease knock-in striatal cell line. Neurol Res 2009; 31: 77–83. 18691453 10.1179/174313208X331572

[bib35] Mojsa B, Mora S, Bossowski JP, Lassot I, Desagher S. Control of neuronal apoptosis by reciprocal regulation of NFATc3 and Trim17. Cell Death Differ 2015; 22: 274–286. 25215946 10.1038/cdd.2014.141 PMC4291489

[bib36] Ventura A, Young AG, Winslow MM, Lintault L, Meissner A, Erkeland SJ et al. Targeted deletion reveals essential and overlapping functions of the miR-17 through 92 family of miRNA clusters. Cell 2008; 132: 875–886. 18329372 10.1016/j.cell.2008.02.019 PMC2323338

[bib37] Pernaute B, Spruce T, Smith KM, Sánchez-Nieto JM, Manzanares M, Cobb B et al. MicroRNAs control the apoptotic threshold in primed pluripotent stem cells through regulation of BIM. Genes Dev 2014; 28: 1873–1878. 25184675 10.1101/gad.245621.114 PMC4197944

[bib38] Bu Q, Fang Y, Cao Y, Chen Q, Liu Y. Enforced expression of miR-101 enhances cisplatin sensitivity in human bladder cancer cells by modulating the cyclooxygenase-2 pathway. Mol Med Rep 2014; 10: 2203–2209. 25109742 10.3892/mmr.2014.2455

[bib39] Zhao X, Wang K, Hu F, Qian C, Guan H, Feng K et al. MicroRNA-101 protects cardiac fibroblasts from hypoxia-induced apoptosis via inhibition of the TGF-β signaling pathway. Int J Biochem Cell Biol 2015; 65: 155–164. 26055514 10.1016/j.biocel.2015.06.005

[bib40] Crow MT, Mani K, Nam YJ, Kitsis RN. The mitochondrial death pathway and cardiac myocyte apoptosis. Circ Res 2004; 95: 957–970. 15539639 10.1161/01.RES.0000148632.35500.d9

[bib41] Kim YM, Kim JH, Kwon HM, Lee DH, Won MH, Kwon YG et al. Korean Red Ginseng protects endothelial cells from serum-deprived apoptosis by regulating Bcl-2 family protein dynamics and caspase S-nitrosylation. J Ginseng Res 2013; 37: 413–424. 24233159 10.5142/jgr.2013.37.413 PMC3825856

[bib42] Min JK, Kim JH, Cho YL, Maeng YS, Lee SJ, Pyun BJ et al. 20(S)-Ginsenoside Rg3 prevents endothelial cell apoptosis via inhibition of a mitochondrial caspase pathway. Biochem Biophys Res Commun 2006; 349: 987–994. 16962070 10.1016/j.bbrc.2006.08.129

[bib43] Liang M, Russell G, Hulley PA. Bim, Bak, and Bax regulate osteoblast survival. J Bone Miner Res 2008; 23: 610–620. 18251703 10.1359/jbmr.080106 PMC2820735

[bib44] Naik E, O’Reilly LA, Asselin-Labat ML, Merino D, Lin A, Cook M et al. Destruction of tumor vasculature and abated tumor growth upon VEGF blockade is driven by proapoptotic protein Bim in endothelial cells. J Exp Med 2011; 208: 1351–1358. 21646395 10.1084/jem.20100951 PMC3135358

[bib45] Kaushal V1, Dye R, Pakavathkumar P, Foveau B, Flores J, Hyman B et al. Neuronal NLRP1 inflammasome activation of Caspase-1 coordinately regulates inflammatory interleukin-1-beta production and axonal degeneration-associated Caspase-6 activation. Cell Death Differ 2015; 22: 1676–1686. 25744023 10.1038/cdd.2015.16 PMC4563782

[bib46] Martinon F, Burns K, Tschopp J. The inflammasome: a molecular platform triggering activation of inflammatory caspases and processing of proIL-β. Mol Cell 2002; 10: 417–426. 12191486 10.1016/s1097-2765(02)00599-3

[bib47] Hagenbuchner J, Ausserlechner MJ. Mitochondria and FOXO3: breath or die. Front Physiol 2013; 4: 147. 23801966 10.3389/fphys.2013.00147 PMC3687139

