# Supplementary material for: A miRNA-101-3p/Bim axis as a determinant of serum deprivation-induced endothelial cell apoptosis
Source: Cell Death Dis. 2017 May 18;8(5):e2808–. doi: 10.1038/cddis.2017.219 (PMC5520733; doi:10.1038/cddis.2017.219)
Supplement: Supplementary Figures [file cddis2017219x1.ppt]

## Slide 1
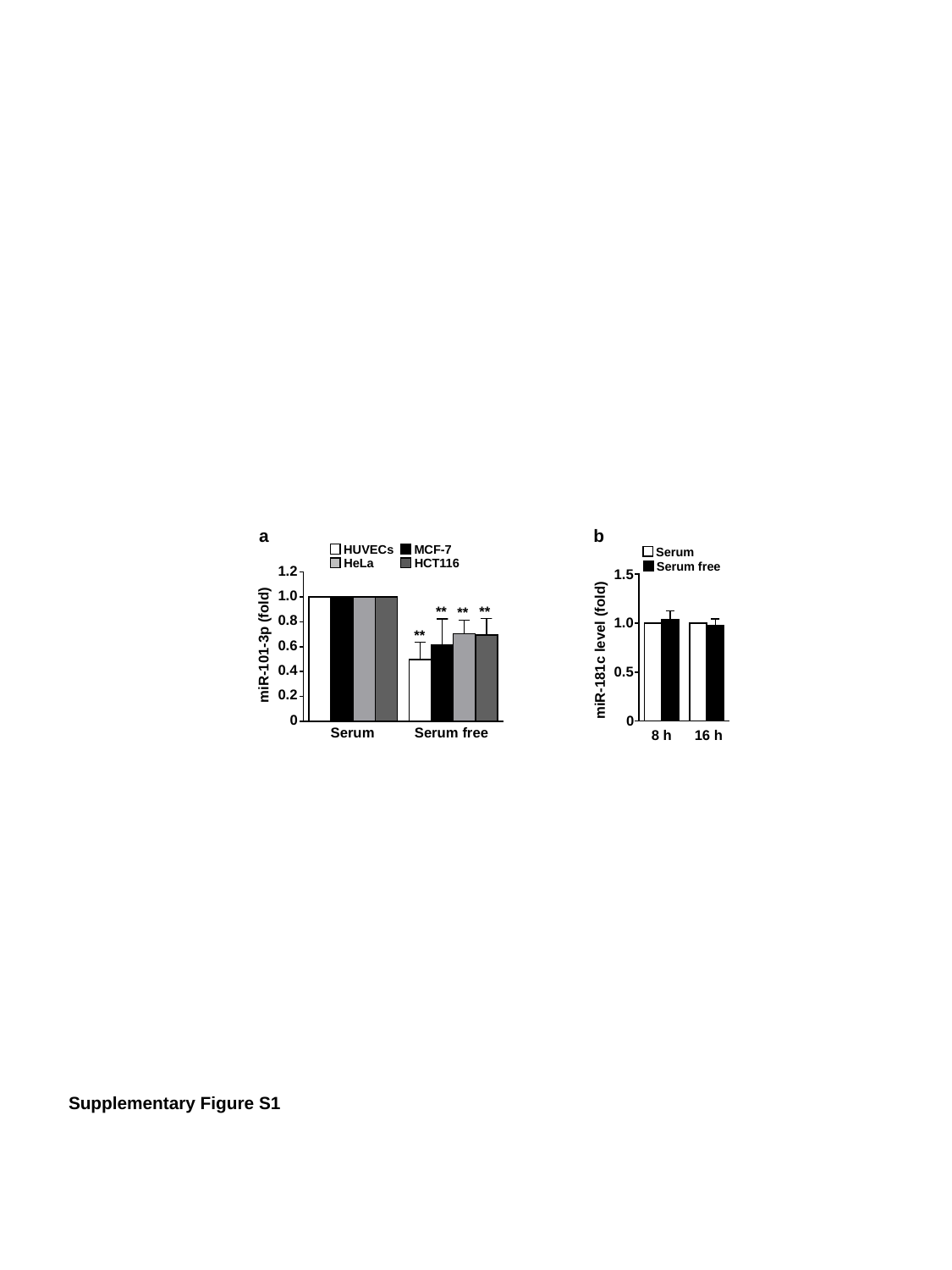

a
b
HUVECs
MCF-7
Serum
HeLa
HCT116
Serum free
1.2
1.5
1.0
**
**
**
0.8
1.0
**
miR-101-3p (fold)
0.6
miR-181c level (fold)
0.4
0.5
0.2
0
0
Serum
Serum free
8 h
16 h
Supplementary Figure S1

## Slide 2
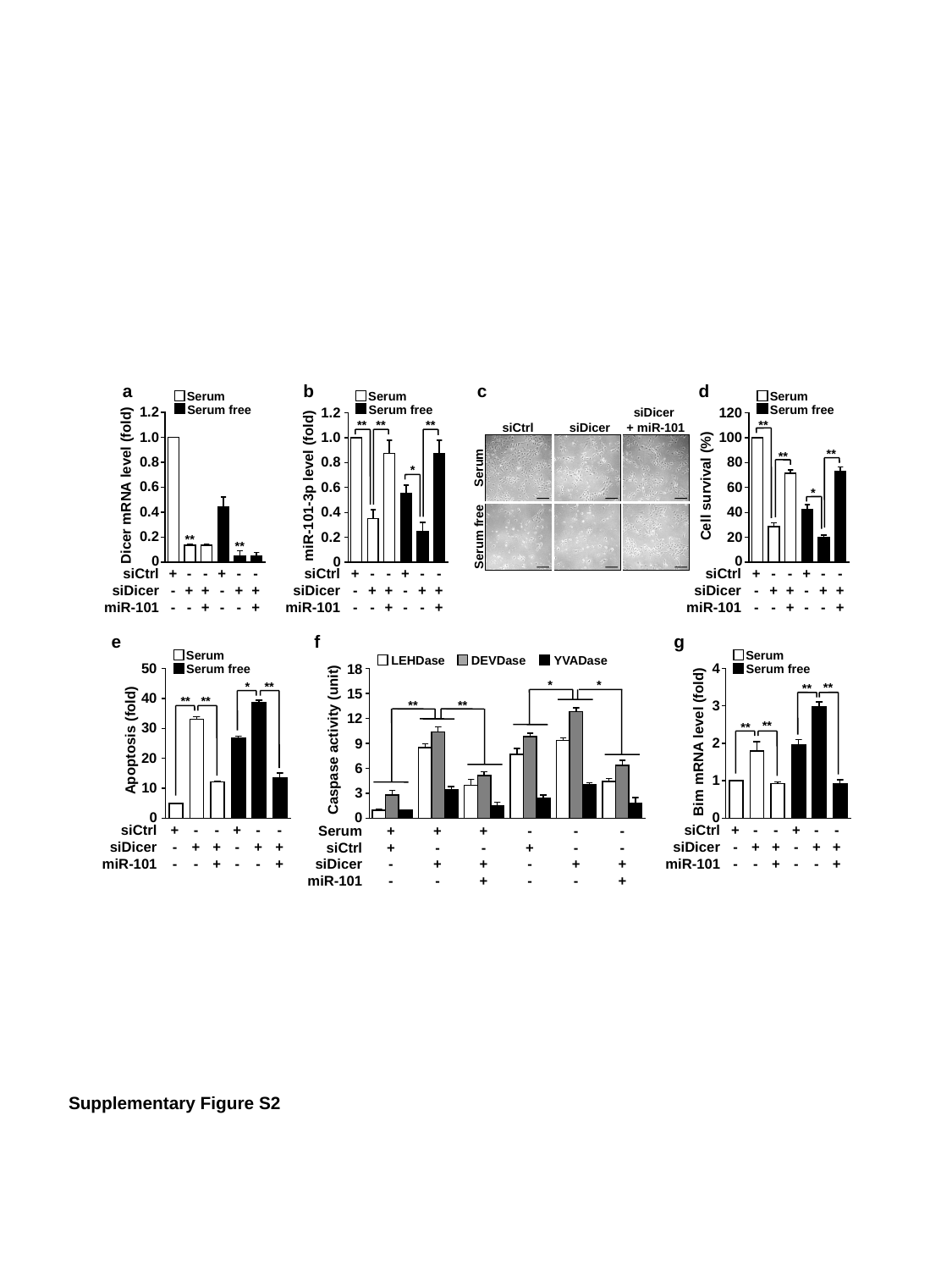

a
b
c
d
Serum
Serum free
Serum
Serum free
Serum
Serum free
1.2
1.2
120
siDicer
+ miR-101
**
**
**
**
siCtrl
siDicer
1.0
1.0
100
**
**
0.8
0.8
80
Serum
*
Dicer mRNA level (fold)
miR-101-3p level (fold)
Cell survival (%)
0.6
0.6
60
*
0.4
0.4
40
0.2
0.2
20
Serum free
**
**
0
0
0
siCtrl
siDicer
miR-101
+
-
-
-
+
-
-
+
+
+
-
-
-
+
-
-
+
+
siCtrl
siDicer
miR-101
+
-
-
-
+
-
-
+
+
+
-
-
-
+
-
-
+
+
siCtrl
siDicer
miR-101
+
-
-
-
+
-
-
+
+
+
-
-
-
+
-
-
+
+
e
f
g
Serum
Serum free
Serum
Serum free
LEHDase
DEVDase
YVADase
50
4
18
*
*
*
**
**
**
15
40
**
**
3
**
**
12
**
30
**
Caspase activity (unit)
Apoptosis (fold)
Bim mRNA level (fold)
2
9
20
6
1
10
3
0
0
0
siCtrl
siDicer
miR-101
+
-
-
-
+
-
-
+
+
+
-
-
-
+
-
-
+
+
siCtrl
siDicer
miR-101
+
-
-
-
+
-
-
+
+
+
-
-
-
+
-
-
+
+
Serum
siCtrl
siDicer
miR-101
+
+
-
-
+
-
+
-
+
-
+
+
-
+
-
-
-
-
+
-
-
-
+
+
Supplementary Figure S2

## Slide 3
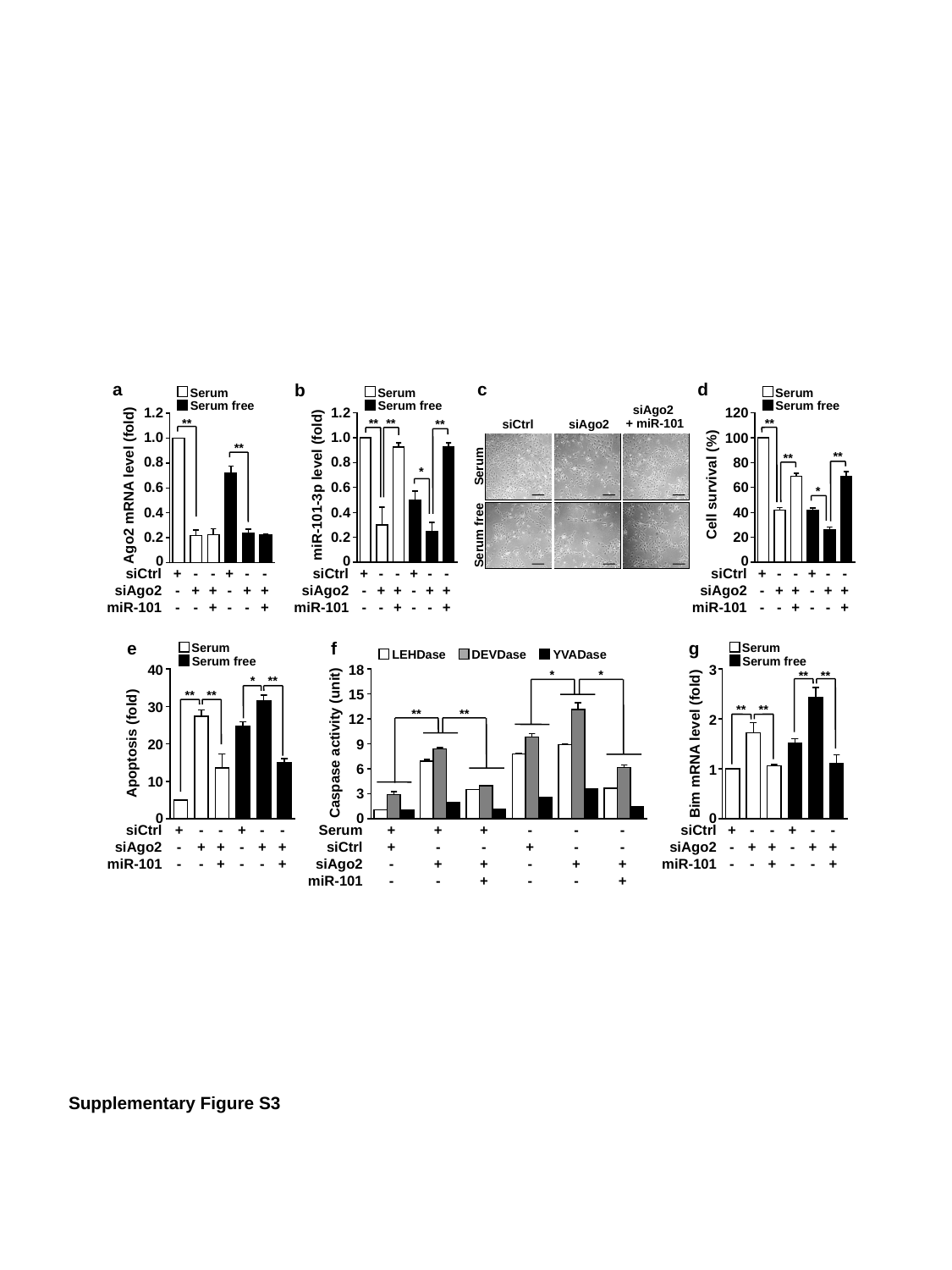

a
c
d
b
Serum
Serum free
Serum
Serum free
Serum
Serum free
siAgo2
+ miR-101
1.2
1.2
120
**
**
**
**
**
siCtrl
siAgo2
1.0
1.0
100
**
**
**
0.8
0.8
80
Serum
*
Cell survival (%)
miR-101-3p level (fold)
Ago2 mRNA level (fold)
0.6
0.6
60
*
0.4
0.4
40
Serum free
0.2
0.2
20
0
0
0
siCtrl
siAgo2
miR-101
+
-
-
-
+
-
-
+
+
+
-
-
-
+
-
-
+
+
siCtrl
siAgo2
miR-101
+
-
-
-
+
-
-
+
+
+
-
-
-
+
-
-
+
+
siCtrl
siAgo2
miR-101
+
-
-
-
+
-
-
+
+
+
-
-
-
+
-
-
+
+
e
f
g
Serum
Serum free
Serum
Serum free
LEHDase
DEVDase
YVADase
40
18
3
*
*
**
**
*
**
15
**
**
30
**
**
**
**
12
2
Caspase activity (unit)
9
Apoptosis (fold)
20
Bim mRNA level (fold)
6
1
10
3
0
0
0
siCtrl
siAgo2
miR-101
+
-
-
-
+
-
-
+
+
+
-
-
-
+
-
-
+
+
Serum
siCtrl
siAgo2
miR-101
+
+
-
-
+
-
+
-
+
-
+
+
-
+
-
-
-
-
+
-
-
-
+
+
siCtrl
siAgo2
miR-101
+
-
-
-
+
-
-
+
+
+
-
-
-
+
-
-
+
+
Supplementary Figure S3

## Slide 4
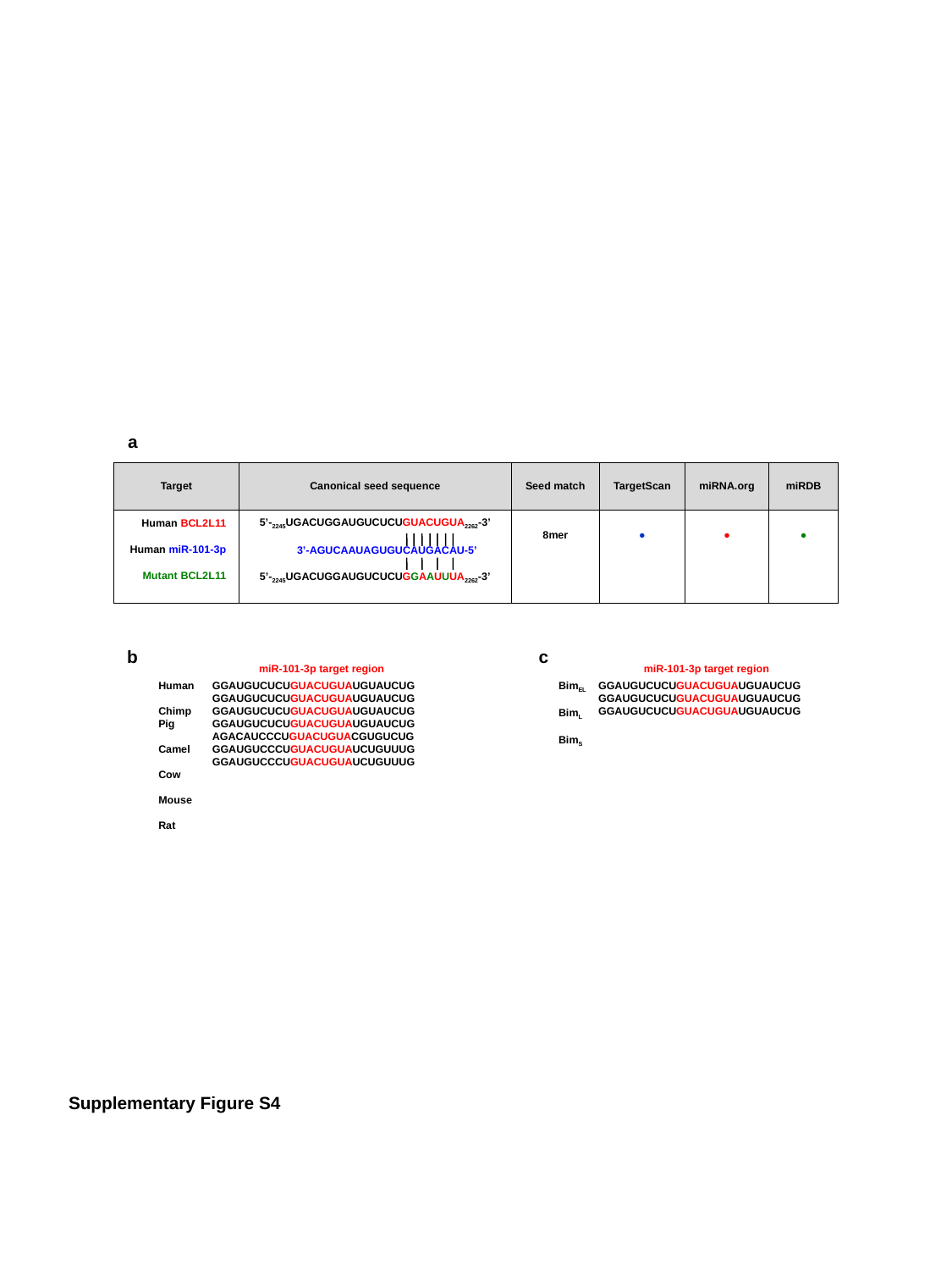

a
| Target | Canonical seed sequence | Seed match | TargetScan | miRNA.org | miRDB |
| --- | --- | --- | --- | --- | --- |
| Human BCL2L11 Human miR-101-3p Mutant BCL2L11 | 5’-2245UGACUGGAUGUCUCUGUACUGUA2262-3’ 3’-AGUCAAUAGUGUCAUGACAU-5’ 5’-2245UGACUGGAUGUCUCUGGAAUUUA2262-3’ | 8mer | ● | ● | ● |
b
c
miR-101-3p target region
Human
Chimp
Pig
Camel
Cow
Mouse
Rat
GGAUGUCUCUGUACUGUAUGUAUCUG
GGAUGUCUCUGUACUGUAUGUAUCUG
GGAUGUCUCUGUACUGUAUGUAUCUG
GGAUGUCUCUGUACUGUAUGUAUCUG
AGACAUCCCUGUACUGUACGUGUCUG
GGAUGUCCCUGUACUGUAUCUGUUUG
GGAUGUCCCUGUACUGUAUCUGUUUG
miR-101-3p target region
BimEL
BimL
BimS
GGAUGUCUCUGUACUGUAUGUAUCUG
GGAUGUCUCUGUACUGUAUGUAUCUG
GGAUGUCUCUGUACUGUAUGUAUCUG
Supplementary Figure S4

## Slide 5
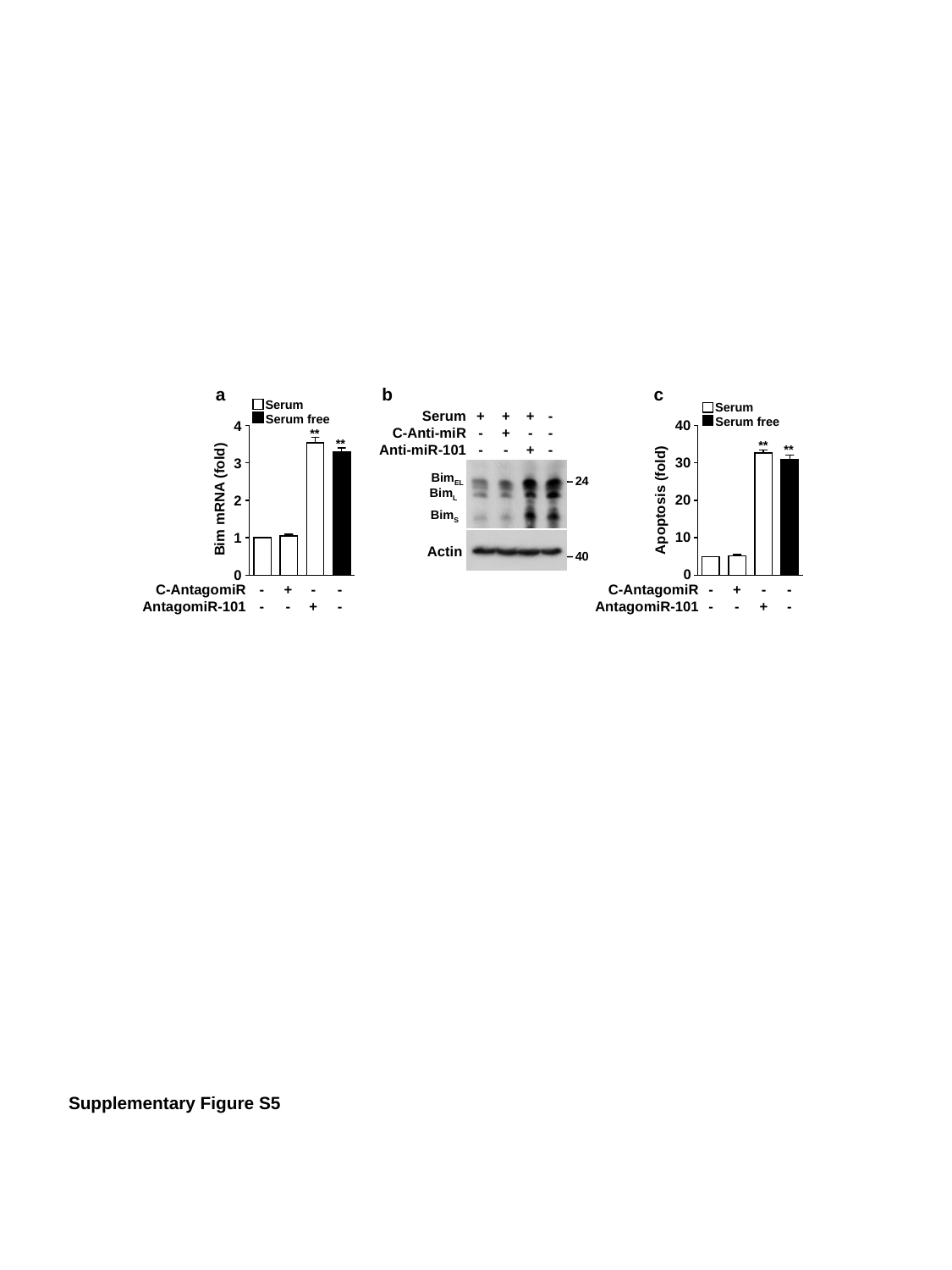

a
b
c
Serum
Serum
Serum
C-Anti-miR
Anti-miR-101
+
-
-
+
+
-
+
-
+
-
-
-
Serum free
Serum free
40
4
**
**
**
**
30
3
BimEL
24
BimL
Bim mRNA (fold)
20
2
Apoptosis (fold)
BimS
10
1
Actin
40
0
0
-
-
C-AntagomiR
AntagomiR-101
-
-
+
-
-
+
-
-
C-AntagomiR
AntagomiR-101
-
-
+
-
-
+
Supplementary Figure S5

## Slide 6
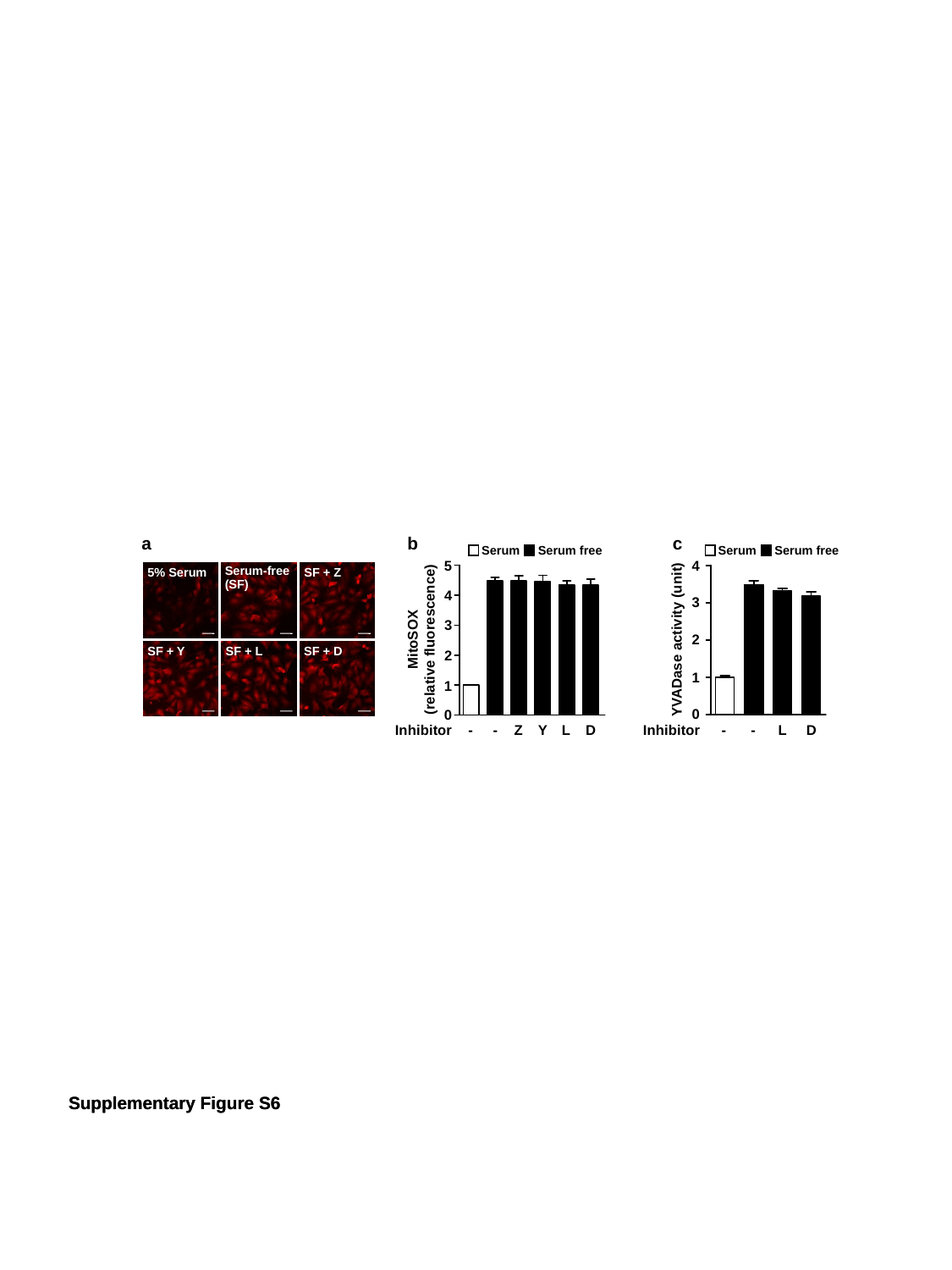

c
a
b
Serum
Serum free
5
4
3
MitoSOX
(relative fluorescence)
2
1
0
Inhibitor
-
-
Z
L
D
Y
Serum
Serum free
4
SF + Z
5% Serum
Serum-free (SF)
SF + Y
SF + L
SF + D
3
2
YVADase activity (unit)
1
0
Inhibitor
-
-
L
D
Supplementary Figure S6
Supplementary Figure S6

## Slide 7
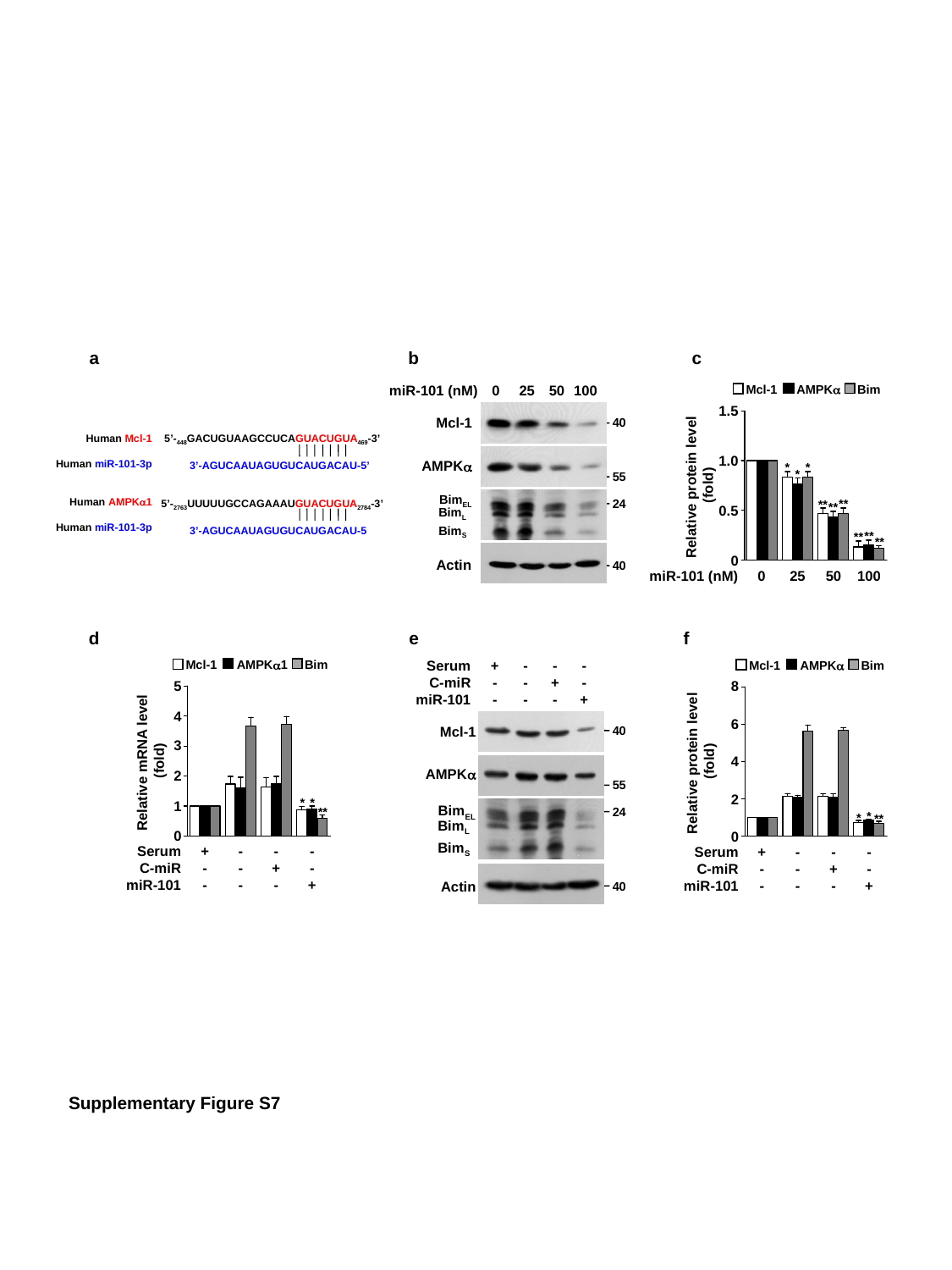

a
b
c
miR-101 (nM)
0
25
50
100
AMPKα
Bim
Mcl-1
1.5
Mcl-1
40
Human Mcl-1
Human miR-101-3p
Human AMPKα1
Human miR-101-3p
5’-448GACUGUAAGCCUCAGUACUGUA469-3’
 3’-AGUCAAUAGUGUCAUGACAU-5’
5’-2763UUUUUGCCAGAAAUGUACUGUA2784-3’
 3’-AGUCAAUAGUGUCAUGACAU-5
1.0
AMPKα
*
*
*
Relative protein level
(fold)
55
BimEL
**
24
**
**
0.5
BimL
BimS
**
**
**
0
Actin
40
miR-101 (nM)
0
25
50
100
d
f
e
AMPKα1
Bim
Mcl-1
Serum
C-miR
miR-101
+
-
-
-
-
-
-
+
-
-
-
+
AMPKα
Bim
Mcl-1
5
8
4
6
40
Mcl-1
3
Relative mRNA level
(fold)
Relative protein level
(fold)
4
AMPKα
2
55
2
*
*
1
BimEL
**
24
*
*
**
BimL
0
0
BimS
Serum
C-miR
miR-101
+
-
-
-
-
-
-
+
-
-
-
+
Serum
C-miR
miR-101
+
-
-
-
-
-
-
+
-
-
-
+
Actin
40
Supplementary Figure S7
