# Supplementary material for: A miRNA-101-3p/Bim axis as a determinant of serum deprivation-induced endothelial cell apoptosis
Source: Cell Death Dis. 2017 May 18;8(5):e2808–. doi: 10.1038/cddis.2017.219 (PMC5520733; doi:10.1038/cddis.2017.219)
Supplement: Supplementary Table S1 [file cddis2017219x2.docx]

**Supplementary Table S1. Sequences of qRT-PCR oligonucleotide primers**

| **Human gene** | **Sense** | **Anti-sense** |
| --- | --- | --- |
| RCL1 | ATGTGATGGCACCACCATTC | GGTGGCAGGGAATATTCAGG |
| Drosha | ACTCCAGTGACCCCATGGAT | GCTTCGTCTTTGGAGGTTCC |
| Dicer | TCTTCGAGCCTCCATTGTTG | CAAATGGTCATCCAGTTCGC |
| Ago2 | AGCAAATTGATGCGAAGTGC | CTTTATTCCTGCCCCCGTAG |
| Bim | CACAAACCCCAAGTCCTCCT | ACACCAGGCGGACAATGTAA |
| GAPDH | GGGGCTCTCCAGAACATCAT | GGTCAGGTCCACCACTGACA |
| NLRP3 | GCCTTGTGACACAGAGGAGC | CCTTCTGCCAGTCAGTGCAG |
| IL-1β | CTCTCCACCTCCAGGGACAG | GAGGCCGATTTCCTTGGTCA |
| IL-18 | TGGCTGCTGAACCAGTAGAAG | GAGGCCGATTTCCTTGGTCA |
| AMPKα1 | TTGTATGCAGGCCCAGAGGT | TGGGATCCACCTGCAGCATA |
| Mcl-1 | AGGAGGACGAGTTGTACCGG | TCCAGTTTCCGAAGCATGCC |
