# Supplementary material for: A miRNA-101-3p/Bim axis as a determinant of serum deprivation-induced endothelial cell apoptosis
Source: Cell Death Dis. 2017 May 18;8(5):e2808–. doi: 10.1038/cddis.2017.219 (PMC5520733; doi:10.1038/cddis.2017.219)
Supplement: Supplementary Figure Legends [file cddis2017219x3.docx]

**Supplementary Figure Legends**

**Supplementary Figure S1**. **Serum deprivation decreases miR-101-3p biogenesis in HUVECs and tumor cells.** HUVECs and tumor cells (MCF-7, HeLa, and HCT116) were cultured in serum-free or 5% FBS-supplemented M199 for 16 h. (**a**) MiR-101 level was determined in tumor cells and HUVECs by qRT-PCR. ***P* < 0.01 vs. serum-stimulated cells. (**b**) MiR-181c level was determined in HUVECs by qRT-PCR.

**Supplementary Figure S2. Dicer knockdown induces apoptosis by increasing Bim expression via downregulation of miR-101-3p biogenesis**. HUVECs were transfected with 100 nM control siRNA (siCtrl), Dicer siRNA (siDicer) or 100 nM miR-101-3p (miR-101), followed by culture in serum-free or 5% FBS-supplemented median for 16 h (gene expression), 24 h (FACS analysis and caspase assay) or 30 h (cell viability). (**a** and **b**) Dicer mRNA and miR-101-3p levels were determined by qRT-PCR. (**c** and **d**) Cell viability was determined by microscopy and MTT assay. Scale bars, 50 μm. (**e**) Apoptosis was evaluated by FACS analysis after staining with Annexin V and PI. (**f**) Caspase activity was determined in cell lysates by colorimetric assay. (**g**) Bim mRNA levels were determined by qRT-PCR. **P* < 0.05 and ***P* < 0.01.

**Supplementary Figure S3**. **Ago2 knockdown induces apoptosis by** **increasing Bim expression via downregulation of miR-101-3p biogenesis**. Cells were transfected with 100 nM siCtrl, Ago2 siRNA (siAgo2) or 100 nM miR-101, followed by culture in serum-free or 5% FBS-supplemented media for 16 h (gene expression), 24 h (caspase assay) or 30 h (cell viability). (**a** and **b**) Levels of Ago2 mRNA and miR-101-3p were determined by qRT-PCR. (**c** and **d**) Cell viability was determined by microscopy and MTT assay. Scale bars, 50 μm. (**e**) Apoptosis was evaluated by FACS analysis after staining with Annexin V and PI. (**f**) Caspase activity was determined in cell lysates by colorimetric assay. (**g**) Levels of Bim mRNA were determined by qRT-PCR. **P* < 0.05 and ***P* < 0.01.

**Supplementary Figure S4. Putative miR-101-3p binding site within the 3’-UTRs of BCL2L11 (Bim) mRNA**. (**a**) Identification of putative complementary sequence for miR-101-3p within human BCL2L11 **(**Bim) mRNA 3′-UTR using TargetScan, microRNA.org, and miRDB. In addition, a mutant sequence of the human Bim 3'-UTR region binding miR-101-3p was shown. (**b**) Putative binding sites of miR-101-3p within the Bim 3′-UTRs of human, non-human primates, and other species. (**c**) Sequence alignment of the 3’-UTRs of human Bim isoforms, which putatively bind miR-101-3p.

**Supplementary Figure S5**. **AntagomiR-101-3p increases Bim expression and apoptosis.** HUVECs were transfected with 100 nM control antagomiR (C-anatomiR) or antagomiR-101-3p, followed by culture in serum-free or FBS-supplemented media in the presence or absence of 50 μM z-VAD-fmk for 16 h (gene and protein expression) or 30 h **(**Annexin V/PI staining)**.** (**a** and **b**) Bim mRNA and protein levels were determined by qRT-PCR and Western blotting. (**c**) Apoptosis was evaluated by Annexin V/PI double staining assay. **P* < 0.05 and ***P* < 0.01 vs. serum-supplemented cells.

**Supplementary Figure S6. Caspase inhibitors do not inhibit ROS production.** Cells were cultured in serum-free or 5% FBS-supplemented media in the presence or absence of 50 μM of z-VAD-fmk (Z), Ac-YVAD-cho (Y), Ac-LEHD-cho (L) or Ac-DEVD-cho (D) for 12 h (ROS assay) or 24 h (casapse assay). (**a**) Mitochondrial ROS generation was determined by MitoSOX-based confocal microscopy. (**b**) Fluorescence intensity was determined by Image J software. (**c**) YVADase activity was determined in cell lysates by colorimetric assay.

**Supplementary Figure S7. Serum deprivation increases Mcl-1 and AMPK expression, but not less effective than Bim expression.** (**a**) Putative binding sequences of miR-101-3p within the 3′-UTRs of human Mcl-1 and AMPKα1. (**b**-**f**) Cells were transfected with C-miR or miR-101 and cultured in fresh media containing 50 μM z-VAD-fmk for 16 h. (**b**, **c**, **e** and **f**) Mcl-1, AMPKα, and Bim protein levels were determined by Western blotting. Relative levels of proteins were determined by Image J software. (**d**) Mcl-1, AMPKα1, and Bim mRNA levels were determined by qRT-PCR.
